# Heteromeric RNP Assembly at LINEs Controls Lineage-Specific RNA Processing

**DOI:** 10.1016/j.cell.2018.07.001

**Published:** 2018-08-23

**Authors:** Jan Attig, Federico Agostini, Clare Gooding, Anob M. Chakrabarti, Aarti Singh, Nejc Haberman, Julian A. Zagalak, Warren Emmett, Christopher W.J. Smith, Nicholas M. Luscombe, Jernej Ule

**Affiliations:** 1 The Francis Crick Institute, Midland Road 1, Kings Cross, London NW1 1AT, UK; 2 Department of Molecular Neuroscience, UCL Institute of Neurology, Queen Square, London WC1N 3BG, UK; 3 Department of Biochemistry, University of Cambridge, Tennis Court Road, Cambridge CB2 1QW, UK; 4 Department of Comparative Biomedical Sciences, The Royal Veterinary College, Royal College Street, London NW1 0TU, UK; 5 Department of Genetics, Environment and Evolution, UCL Genetics Institute, Gower Street, London WC1E 6BT, UK; 6 Okinawa Institute of Science and Technology Graduate University, 1919-1 Tancha, Onna-son, Kunigami-gun, Okinawa 904-0495, Japan

**Keywords:** splicing, alternative polyadenylation, LINE repeats, multivalency, CLIP, MATR3, PTBP1, evolution, cryptic exons, exonogenesis

## Abstract

Long mammalian introns make it challenging for the RNA processing machinery to identify exons accurately. We find that LINE-derived sequences (LINEs) contribute to this selection by recruiting dozens of RNA-binding proteins (RBPs) to introns. This includes MATR3, which promotes binding of PTBP1 to multivalent binding sites within LINEs. Both RBPs repress splicing and 3′ end processing within and around LINEs. Notably, repressive RBPs preferentially bind to evolutionarily young LINEs, which are located far from exons. These RBPs insulate the LINEs and the surrounding intronic regions from RNA processing. Upon evolutionary divergence, changes in RNA motifs within LINEs lead to gradual loss of their insulation. Hence, older LINEs are located closer to exons, are a common source of tissue-specific exons, and increasingly bind to RBPs that enhance RNA processing. Thus, LINEs are hubs for the assembly of repressive RBPs and also contribute to the evolution of new, lineage-specific transcripts in mammals.

**Video Abstract:**

## Highlights

• Multivalent binding sites in young LINEs mainly recruit repressive RBPs
• PTBP1 and MATR3 co-bind and repress RNA processing within and around young LINEs
• 1-kb exclusion zone of LINEs from vicinity of exons reflects their repressive effects
• Sequence-driven RBP remodeling on old LINEs drives evolution of tissue-specific exons

## Graphical Abstract

**Figure:**
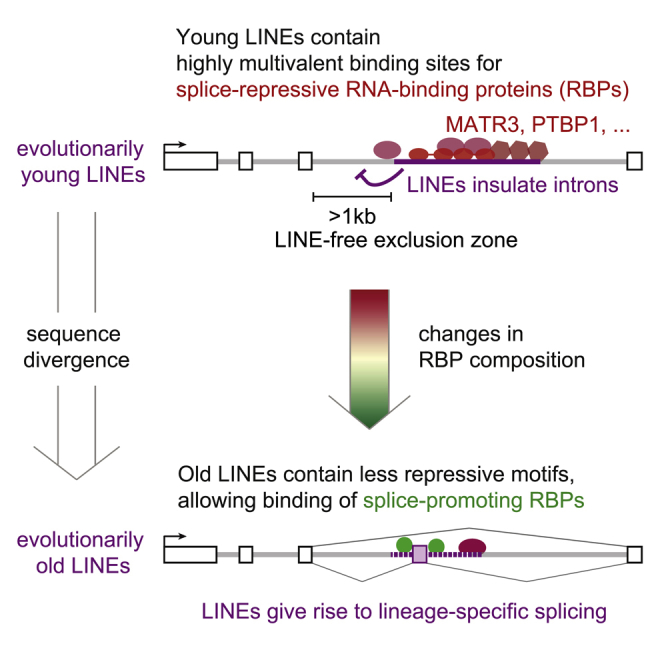

## Teaser

LINE elements recruit RNA-binding proteins to mammalian introns, influencing splicing and playing a key role in the evolution of tissue-specific exons.

## Introduction

Human introns are replete with sequences that resemble splice sites and poly(A) sites, creating a demand for mechanisms to help the RNA processing machinery distinguish true from so-called cryptic RNA processing sites. Inappropriate recognition of such sites initiates inclusion of cryptic exons, which can disrupt gene expression by changing the reading frame, introducing premature stop codons, and decreasing transcript stability. Several RNA-binding proteins (RBPs) are known to contribute to splicing fidelity by repressing cryptic splice sites (Sibley et al., 2016), but identification of RBPs that repress cryptic RNA processing sites remains anecdotal.

The human genome contains more than 1.4 million fragments of LINE repeats, many of which are located in introns (Smit, 1999). The two most common LINE repeat families in mammals are L1 and L2. Active L1 contains its own promoter in the 5′ UTR and encodes for two open reading frames, ORF1p and ORF2p (Feng et al., 1996, Burns and Boeke, 2012), as a bicistronic mRNA. Active L1s are ∼6 kb long (such as L1HS) (Smit et al., 1995) although individual families differ largely in their 5′ and 3′ UTRs and can be substantially longer, such as the L1MA2 family with a size of ∼7.6 kb. In the human genome, only ∼250 L1 insertions encode a functional ORF2p (Penzkofer et al., 2017) and only 60–80 account for all *de novo* LINE insertions observed in human populations or *in vitro* (Beck et al., 2010, Brouha et al., 2003). The remaining L1 and all L2 elements are mostly degenerated and truncated compared to the families’ consensus sequences, and mutations have disrupted their ability to retrotranspose. In spite of their prevalence, the effects of intragenic LINEs on splicing have been studied mainly in individuals with hereditary diseases, where an intronic LINE insertion disrupts expression of an individual gene, such as CYBB (Meischl et al., 2000), DMD (Yoshida et al., 1998), and XRP2 (Schwahn et al., 1998). Several RBPs, such as UPF1, ELAVL1, and ZCCHC3, are known to bind active LINEs and thereby interfere with their retrotransposition (Goodier et al., 2013, Taylor et al., 2013). However, the regulatory potential of intronic LINEs, and the RBPs binding them, are poorly characterized.

Here, we surveyed iCLIP and eCLIP data to identify 28 RBPs with enriched binding to LINEs, including MATR3 and PTBP1. MATR3 promotes binding of PTBP1 to LINEs at “multivalent binding sites,” composed of multiple short binding motifs that are clustered together. The two RBPs jointly block the recognition of cryptic poly(A)-sites and splice sites within LINEs. We demonstrate that evolutionarily recent L1 elements recruit repressive RBPs to introns, while many evolutionarily older LINEs have partially escaped from this repression and contribute to the emergence of exons specific to the mammalian lineage. Thus, we link the functional relevance of LINEs to dozens of interacting RBPs and demonstrate the importance of combinatorial binding of RBPs to repetitive elements, exemplified by MATR3 and PTBP1.

## Results

### LINE-Derived Sequences Recruit Dozens of RBPs to Deep Intronic Regions

**Figure 1:**
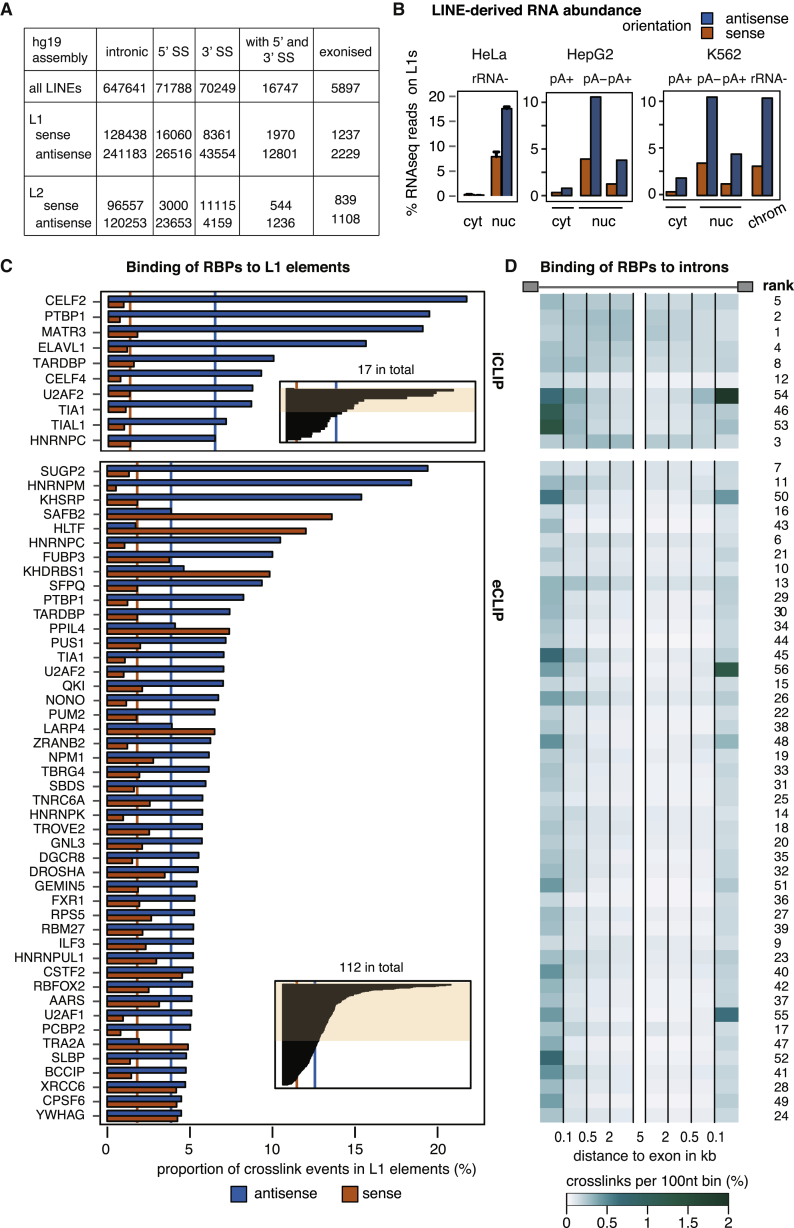
LINEs Are Binding Platforms for Diverse RBPs (A) Number of LINE fragments within introns of human genes based on UCSC annotation (hg19 assembly), the number of LINEs with a 3′ or 5′ splice sites and the number of LINEs forming an exon. The total number of exonized elements is given, which includes elements contributing a poly(A) termination site to a terminal exon in addition to those contributing a 3′ or 5′ splice site. (B) Estimate of abundance of L1-sequences in subcellular RNA fractions from HeLa, K562, and HepG2 cells. Strand-specific RNA-seq was used to quantify abundance of L1 in sense and antisense (orange and blue), relative to the number of mapped reads. Data is split for libraries made from polyA−, polyA+, or rRNA− RNA. Data for K562 and HepG2 is from the ENCODE consortium. Data for HeLa is from triplicates and is shown as mean ± SD. cyt, cytoplasmic RNA; nuc, nuclear RNA; chrom, chromatin-associated RNA. (C) Frequency of L1 repeat sequences among the bound RNA sequences of a panel of RBPs. Because e/iCLIP is strand-specific, binding to LINEs transcribed in sense or in antisense was quantified separately (orange and blue). Orange and blue lines indicate median binding across all RBPs. The inlet indicates the section of the full dataset shown, the full dataset including sources is available in Table S2. For visualization, replicates were averaged and only data from one cell line is shown. (D) Binding to introns of at least 7 kb size was analyzed in 100-nt bins up to 5 kb upstream and downstream of the exon and quantified in percent relative to the total number of mapped reads. Data is shown for the first 100-nt bin and as an average of the 100-nt windows within 101–500 nt, 501–2,000 nt, and 2,001–5,000 nt distance. A rank for deep intronic binding is given based on the average of the first 100 nt of either splice site and average binding in the 2,001- to 5,000-nt window. See also Figure S2 and Table S2.

According to RepeatMasker annotation, the human genome contains ∼1.4 million fragments of LINE repeats, ∼650,000 located in introns (Figure 1A) (Smit, 1999). These intronic LINEs often contain splice site sequences, but rarely give rise to exons according to public exon annotation. We therefore wished to study if repressive RBPs prevent the use of cryptic processing sites at LINEs. The abundance of LINE-derived sequences in pre-mRNA is reflected in bulk sequencing of nuclear, but not cytoplasmic RNA, in HeLa, K562, and HepG2 cell lines (Figure 1B). To identify RBPs that bind to L1-derived sequences, we examined iCLIP data for 17 RBPs and eCLIP data from K562 and HepG2 cells for 112 RBPs available from ENCODE (Table S1) (Sloan et al., 2016, Van Nostrand et al., 2017). We ranked these RBPs by the proportion of crosslink events mapping to sense or antisense L1 elements (Figure 1B).

**Figure S1:**
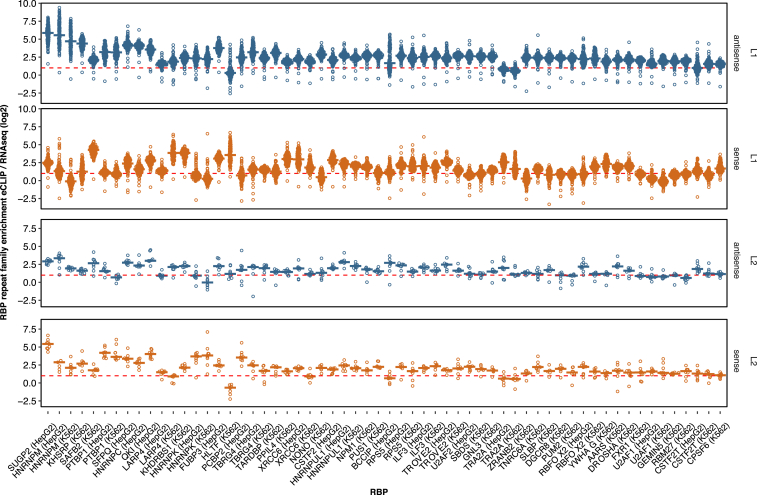
Extended Data for LINEs Are Binding Platforms for a set of RBPs, Related to Figure 1 TEtranscript (Jin et al., 2015) was used to estimate the enrichment of each subfamily of L1 and L2 repeats among the bound RNA sequences of a panel of RBPs, comparing the abundance in recovered eCLIP tags to the abundance in RNaseq reads. For each RBP, all 142 L1/L2 subfamilies (132 for L1, 10 for L2) were considered. Since eCLIP is strand-specific, binding to LINEs transcribed in sense or in antisense were quantified separately, colored in red and blue. The cell lines used in each eCLIP experiment are indicated on the bottom.

CELF2, MATR3, and PTBP1 ranked highest in our iCLIP data and SUGP2, HNRNPM, and KHSRP in the eCLIP data (Figure 1C; Table S2). For PTBP1, enrichment on antisense L1s is confirmed by the eCLIP data (that is not available for CELF2 and MATR3) and by a previous study that compared PTBP1 iCLIP reads in LINEs to a genomic null model (Kelley et al., 2014). Dozens of additional RBPs had enriched binding on L1 elements in i/eCLIP data, with antisense orientation being most commonly bound. We also examined RBP binding to L2 elements, which are approximately three times less common in the human genome than L1s. Over a dozen RBPs were enriched on L2s in a strand-specific manner, with SUGP2, MATR3, PTBP1, and HNRNPK showing strongest enrichment in sense L2s, and HNRNPA1, TAF15, HNRNPU, and SAFB2 in antisense L2s (Table S2). Genomic mapping of sequencing reads partially discards highly repetitive sequences, so we also examined eCLIP RBP binding to sub-families of LINEs by using the TEtranscripts method (Jin et al., 2015), which recapitulated our ranking (Figure S1). Thus, in spite of the repetitive nature of LINE sequences, most are divergent enough to enable unique genomic mapping of CLIP reads. In total, 25 RBPs had more than 2-fold enrichment on L1 or L2 elements according to TEtranscripts, which together with MATR3, CELF2, and ELAVL1 that are more than 2-fold enriched by iCLIP, identifies 28 LINE-binding RBPs.

Finally, we assessed the distribution of intronic binding sites of LINE-binding RBPs relative to exons (Figure 1D). Interestingly, we find that positional preferences of most RBPs are either skewed toward the first 500 nt next to exons, or “deep intronic” regions, those more than 500 bp away from any annotated exon. We ranked the RBPs according to the binding pattern, which shows that LINE-binding RBPs often preferentially bind to deep intronic regions. This is most apparent for MATR3 and PTBP1, which ranked highest as deep intronic binders.

### MATR3 Stabilizes Multivalent PTBP1-RNA Binding, Especially on L1s

**Figure S2:**
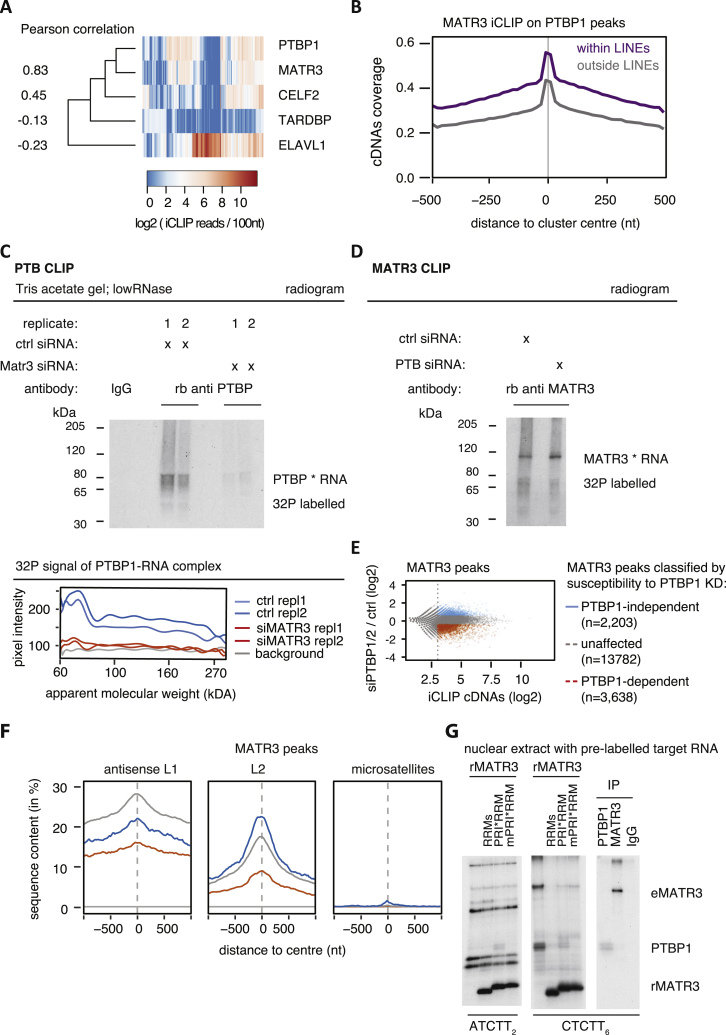
Combinatorial Binding of MATR3 and PTBP1 to the Same LINEs, Related to Figure 2 (A) For each RBP that showed considerable binding to LINE repeats in iCLIP (see B), we selected the 50 LINE repeats with strongest coverage (cDNAs per 100nt). For comparison we included TARDBP, which showed little binding to LINE repeats. All iCLIP data selected was collected from HEK293 cells. The heatmap shows comparison of binding strength at this set of 214 LINE repeats, and the nearest neighbor analysis for each RBP. The values left to the dendrogram show the Pearson correlation coefficient between all RBPs and PTBP1. Only LINEs with a minimal length of 50nt were considered to reduce the bias to short, highly expressed LINE repeats. (B) Metaprofile of iCLIP binding for MATR3 around iCLIP binding peaks of PTBP1 within and outside of LINE repeats. The data was smoothed with 20nt bins. (C) HEK293T cells were transfected with siRNAs targeting MATR3, PTBP1 or scrambled controls, and 72 hours later labeled with 100μM 4SU for 8 hours and cross-linked with 365nm UV light. The radiogram shows ^32^P labeled RNA crosslinked to and co-precipitated with PTBP1. Before immunoprecipitation, protein concentration was measured and equalised. The PTBP1 iCLIP was done under low RNase conditions (compare with Figure 2A for high RNase condition). Replicate 1 and 2 are independent biological replicates processed in parallel. (D) ^32^P labeled RNA crosslinked to and co-precipitated with MATR3 under equivalent conditions as in (C). The MATR3 iCLIP shown was done under high RNase conditions. (E) MATR3 binding peaks were identified from iCLIP experiments, and classified according to susceptibility to PTBP1 depletion as indicated based on moderated log2 fold change. Binding peaks with a normalized count of less than 8 were ignored, as indicated by the dotted line. (F) The overlap between the center of MATR3 binding peaks and different repeat classes was tested for antisense L1 elements, sense L2 elements, and sense CT-/T-rich microsatellite repeats. Metaprofiles show the percentage of each class of clusters overlapping with each genomic element, and PTBP1-dependent and –independent MATR3 binding peaks are color-coded as in (E). (G) Protein-protein interaction between MATR3 and PTBP1 allows recruitment of PTBP1 to a MATR3 bound RNA *in vitro*. Recombinant MATR3 mutants (rMATR3) and 32P labeled RNA probes were added to nuclear extracts from HeLa cells and UV-crosslinked. RNA substrates contained either two MATR3 or six PTBP1 RNA compete motifs motifs (ATCTT_2_ and CTCTT_6_). Crosslinking signals corresponding to endogenous PTBP1 (PTBP1) and MATR3 (eMATR3) were confirmed by immunoprecipitation.

**Figure 2:**
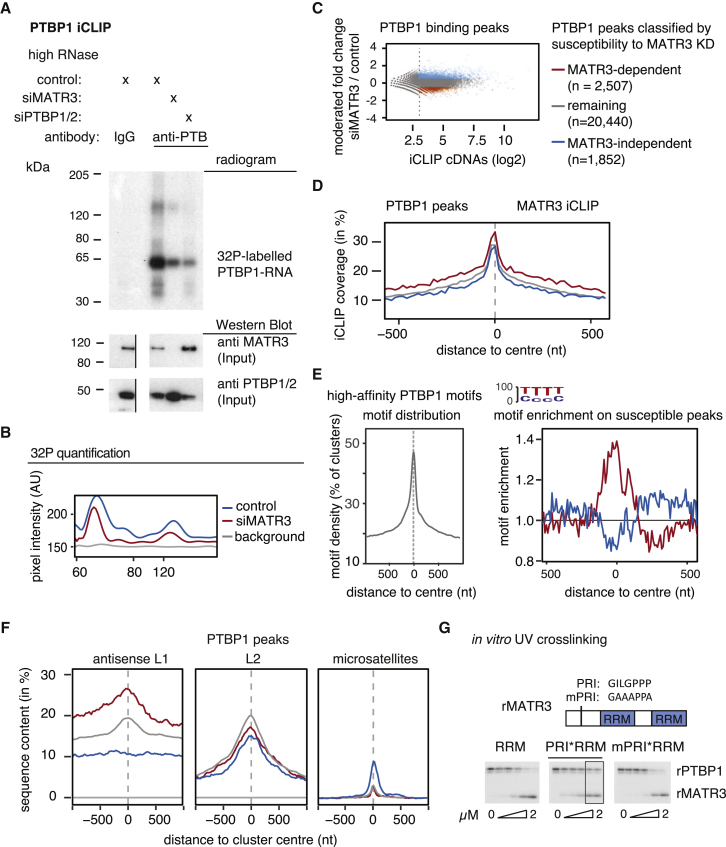
Binding of PTBP1 to Antisense L1 Elements Is MATR3-Dependent PTBP1 iCLIP was performed from HEK293T cells depleted of MATR3 as well as controls. MATR3-dependent PTBP1 binding clusters are shown in red and MATR3-independent PTBP1 binding clusters in blue (C–F). (A) RNA crosslinked to and co-precipitated with PTBP1 under high RNase conditions was labeled with ^32^P-ATP; the size of the PTBP1-RNA is marked next to the radiogram gel image. The input lysate for the iCLIP experiment was probed for MATR3 and PTBP1 antibodies in a western blot. The gel image was cut to align it with the radiogram. Replicates are shown in Figure S2A, and Figure S3C shows another western blot assessing MATR3 and PTBP1 protein levels in the relevant conditions. (B) To quantify the signal, gray pixel intensity measured across the center of each lane is shown, analyzed with ImageJ software. (C) PTBP1 binding peaks were identified from all iCLIP experiments and classified according to their susceptibility to MATR3 depletion. Binding peaks with a normalized count of <8 were ignored, indicated by the dotted line. (D) Coverage of MATR3 iCLIP around MATR3-dependent PTBP1 binding peaks. (E) Enrichment for high-affinity PTBP1 binding motifs around PTBP1 binding peaks. Left: all PTBP1 binding peaks show strong enrichment for PTBP binding motifs. Right: MATR3-dependent PTBP1 binding peaks show enrichment in a 200-nt region for high-affinity motifs above other PTBP1 binding peaks. (F) The overlap between the center of PTBP1 binding peaks and different repeat classes was tested for antisense L1 elements, sense L2 elements, and sense CT-/T-rich microsatellite repeats. Metaprofiles show the percentage of each class of clusters overlapping with each genomic element. (G) Protein-protein interactions between MATR3 and PTBP1 allow the formation of a heteromeric complex on a substrate RNA with two ATGTT motifs *in vitro*. Recombinant PTBP1 (rPTBP1) and different MATR3 mutants (rMATR3) were crosslinked to the same RNA at different MATR3 molarity (rPTBP1 at 0.5 μM).

MATR3 directly interacts with PTBP1 (Coelho et al., 2015), but it is not known if this affects their RNA binding specificity. Unsupervised clustering of LINEs bound by MATR3, PTBP1, TARDBP, ELAVL1, and CELF2 showed the strongest correlation between MATR3 and PTBP1 (Pearson coefficient = 0.83, Figure S2A). Moreover, MATR3 binding was enriched in the proximity of PTBP1 binding peaks, with a further increase within LINEs (p value < 2.2e−16, Figure S2B). Therefore, we examined if MATR3 and PTBP1 are dependent on each other for binding to LINEs by performing iCLIP with PTBP1 in HEK293 cells depleted of MATR3, and iCLIP with MATR3 in HEK293 cells depleted of PTBP1 and PTBP2 (PTBP1/2), as well as cells transfected with control small interfering RNA (siRNA) (Figures 2A, S2C, and S2D). Notably, we immunoprecipitated a decreased amount of RNA crosslinked to PTBP1 upon MATR3 depletion, as measured by ^32^P labeling, which was not fully explained by a change in the abundance of PTBP1 protein (Figure 2A; replicates in Figure S2C). Conversely, the amount of RNA crosslinked to MATR3 did not change upon depletion of PTBP1/2 (Figure S2D).

Next, we classified the peaks of PTBP1 crosslinking into MATR3-dependent, MATR3-independent, and remaining peaks (Figure 2C). As expected, all crosslinking peaks were highly enriched for CT-rich motifs, most prominently at the peak center (Figure 2E). Importantly, MATR3-dependent PTBP1 peaks better overlapped with MATR3 crosslinking than MATR3-independent peaks (Figure 2D) and had a higher overall density of CT-rich motifs over a 200-nt region around the peak (Figure 2E). We also examined the overlap of PTBP1 peaks with genomic repeats. MATR3-dependent PTBP1 peaks were more strongly enriched in antisense L1 elements compared to the remaining peaks (Figure 2F). Conversely, PTBP1 is not required for MATR3 binding to LINEs (Figures S2D–S2F). PTBP1 also binds CT- and T-rich microsatellite repeats (Ling et al., 2016), but this accounts for only ∼0.2% of all PTBP1 peaks in unperturbed HEK293 cells, and they are only found within MATR3-independent peaks. This suggests that MATR3 supports the binding of PTBP1 to the most multivalent binding sites (i.e., sites that contain multiple CT-rich motifs that are highly clustered over a region that can span up to 200 nt around the binding peak). Such sites are particularly frequent within antisense L1 elements.

To further examine how MATR3 affects binding of PTBP1 to RNA, we used *in vitro* binding assays. We previously found a PTBP1 RRM2 interacting (PRI) motif within the disordered region of MATR3, which is essential for interaction with PTBP1 RRM2 (Coelho et al., 2015). We purified recombinant MATR3 fragments (rMATR3) comprising its two RRMs, with (“PRI-RRMs”) or without the PRI motif (“RRMs”), or with mutations within the PRI that abolishes PTBP1 binding (“mPRI-RRMs”). We designed an *in vitro* synthesized RNA with two MATR3 RNAcompete motifs (ATCTT) (Ray et al., 2013) as well as small CT-stretches, which allowed binding of either MATR3 or PTBP1 (Figure 2G). Notably, the non-interacting rMATR3 (RRMs or mPRI-RRMs) competed with PTBP1 for RNA binding at equimolar concentrations (Figure 2G), but the interacting PRI-RRM rMATR3 enabled PTBP1 crosslinking even when rMATR3 was present at excess molarity. We also added rMATR3 to HeLa nuclear extracts with endogenous PTBP1 and assayed binding to an RNA probe containing two ATCTT motifs (as before), or a probe with a multivalent binding site containing six CTCTT motifs (the RNAcompete motif for PTBP1) (Figure S2G). Again, addition of an excess of the non-interacting rMATR3 (RRMs or mPRI-RRMs) prevented PTBP1 crosslinking to both RNAs, while addition of the interacting PRI-RRM rMATR3 increased crosslinking to ATCTT_2_, and preserved crosslinking to CTCTT_6_ RNA. It is likely that the PRI-motif allows the formation of a MATR3/PTBP1/RNA complex, and this promotes the *in vitro* binding of PTBP1 to multivalent binding sites.

### MATR3 and PTBP1 Co-repress LINE-Derived Exons and Poly(A) Sites

**Figure 3:**
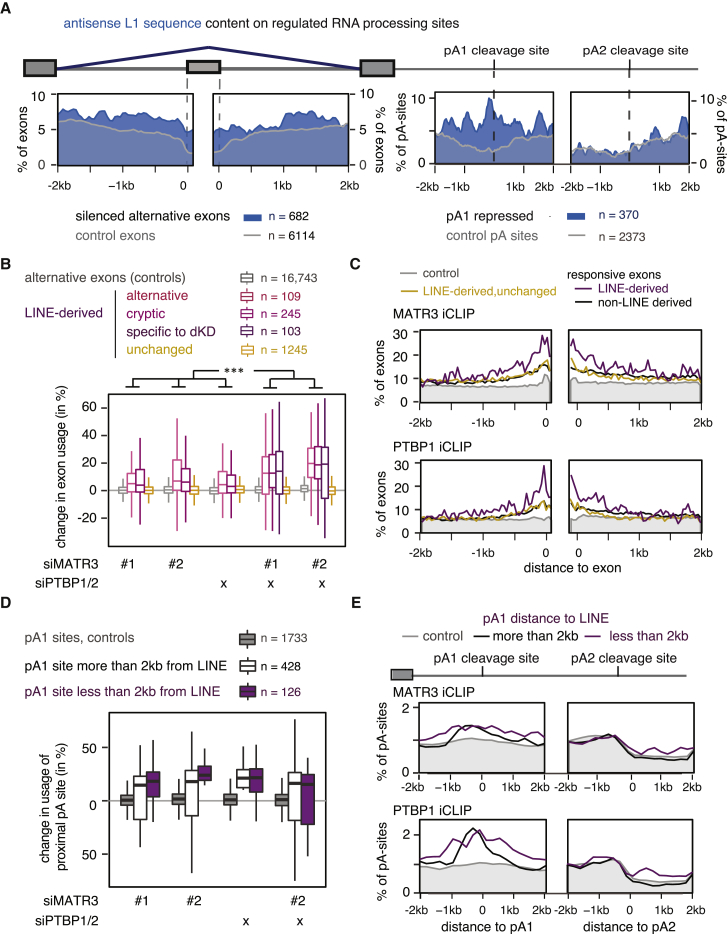
MATR3 and PTBP1 Repress Splice and Poly(A) Sites in LINEs (A) The metadata profile shows the coverage of antisense L1 sequences in a ±2 kb window flanking the splice sites and the proximal and distal poly(A) sites of MATR3/PTBP1/2 repressed events or control. Metadata profile was smoothed using 40-nt bins. (B) LINE-derived exons were identified *de novo* from RNA-seq data of HeLa cells depleted of MATR3 and PTBP1. Differences in exon inclusion across groups were tested by Kruskal-Wallis rank-sum test (p value < 2.2e^−16^) and pairwise comparisons by Dunn’s test corrected according to Holm-Šidák. ^∗∗∗^Adjusted p value < 0.001 in all indicated comparisons. LINE-derived exons specific to the MATR3/PTBP1 depleted condition were of too low read count for quantification in the other conditions. (C) Metadata profiles of MATR3 and PTBP1 iCLIP binding across ±2 kb of the splice site of LINE-derived exons shown in (B). iCLIP binding is presented as a percentage of occupancy, and was smoothed using 40-nt bins. Occupancy on non-regulated sites is shown in gray as control. (D) Percent change in the use of the proximal poly(A) sites. poly(A) sites are split into those within 2 kb vicinity of a LINE and those that are not. (E) Metadata profiles of MATR3 and PTBP1 iCLIP binding as in (C) across ±2 kb of the poly(A) sites shown in (D). See also Figures S3 and S4 and Tables S3 and S4.

**Figure S3:**
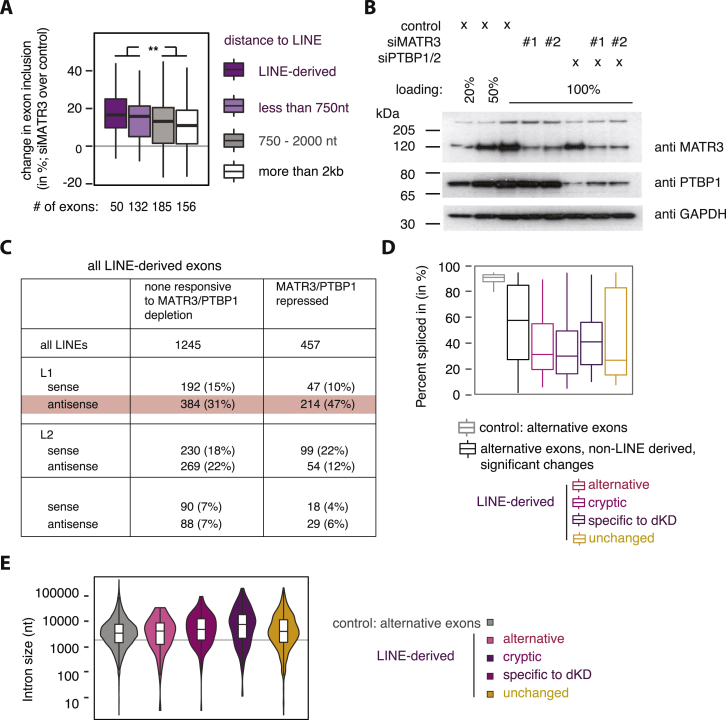
Features of LINE Elements Repressed by MATR3 and PTBP1, Related to Figure 3 (A) Established alternative exons derived from or within 750nt to a LINE are more strongly repressed by MATR3 than those that are further away. The differences in repression strength across groups was tested by Kruskal-Wallis Rank Sum test (across all four conditions p value = 0.0193; comparison as indicated p value = 0.00335). (B) Semiquantitative western blot showed efficient depletion of MATR3 and PTBP1 in cells transfected with siRNAs against MATR3 or PTBP1/2 individually or in combination. (C) The class and orientation of the LINEs that seed exons repressed by MATR3/PTBP1. (D) Percent exon inclusion estimates of LINE-derived exons in unperturbed HeLa cells. Exons are grouped as in Figure 3B. (E) MATR3/PTBP1 repressed LINE-derived exons are within long introns. Intron size is the total distance between the flanking exons. The gray line indicates an intron length of 2kb.

Given the coordinated binding of MATR3 and PTBP1 to LINEs, we wished to understand the functional importance of this binding. First, we re-analyzed our previous splice junction microarray data (Coelho et al., 2015) and found ∼2-fold enrichment of antisense L1 sequence overlapping exons repressed by MATR3 or PTBP1 and enrichment extended for up to 2 kb around the exons (Figure 3A). We also observed that the distance of MATR3-repressed exons from antisense L1s anti-correlates with the strength of repression (Figure S3A). Next, we performed RNA sequencing (RNA-seq) from HeLa cells depleted of MATR3 and PTBP1/2, individually or in combination and used *de novo* transcriptome assembly to identify cryptic exonization events. We detected 1,702 LINE-derived exons in total; 1,180 of which are not identical with UCSC exon annotation and can therefore be considered as cryptic exons (Table S3). Depletion of both MATR3 and PTBP1/2 led to the differential use of 457 (∼27%) of all detected LINE-derived exons, the great majority of which are de-repressed (Figure 3B). Repression of LINE-derived exons by MATR3 and PTBP1 was additive, as evident by the strongly increased inclusion upon their co-depletion. We found an enrichment for antisense L1 elements among MATR3/PTBP1/2 repressed LINE-derived exons (Figure S3C), and they were preferentially located within long introns (Figure S3E). Thus, MATR3 and PTBP1 are primarily repressing exons emerging from deep intronic L1 elements. Metaprofiles of iCLIP data showed increased binding of MATR3 and PTBP1 around the significantly repressed LINE-derived exons, confirming their direct regulation (Figure 3C).

**Figure S4:**
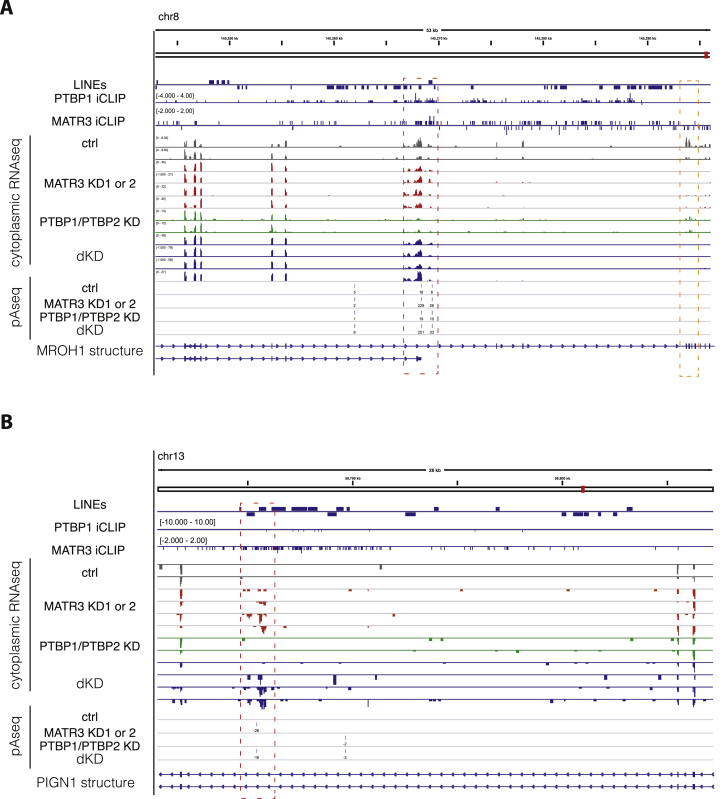
Emergence of New Termination Sites following MATR3/PTBP1 Depletion, Related to Figure 3 Examples of MATR3/PTBP1 repressed poly(A) sites. Genome browser tracks show position and orientation of LINE insertion (hg19/RepeatMasker annotation), PTBP1 and MATR3 iCLIP coverage, as well as tracks for RNaseq of cytoplasmic RNA and mRNA 3′ end sequencing (pA-seq) from total RNA. All tracks are scaled appropriately to library size. (A) The *MROH1* gene shows inclusion of additional exonic sequence and two different terminal exon isoforms in MATR3 depleted cells (highlighted by red dashed lines). Inclusion of this alternative terminal exon appears to cause premature transcriptional termination, as seen by loss of expression downstream of the exon (highlighted by orange dashed lines). (B) Use of a cryptic processing site in the *PIGN1* results in a new exon and a new poly(A) site, derived from two antisense L1 insertions (highlighted by red dashed lines).

We also produced 3′ end sequencing data to investigate the regulation of poly(A) sites, because antisense L1 elements are rich in cryptic poly(A)-signals (Han et al., 2004, Lee et al., 2008). We used the expressRNA platform (Rot et al., 2017) to find 5,189 genes with two poly(A) sites, each containing at least 5% of the sequencing reads within the gene (referred to as pA1 and pA2). Of these, 240 poly(A) sites originated from a LINE (Table S4). LINEs were enriched at proximal poly(A) sites repressed by MATR3/PTBP1 and up to ∼2 kb away from the sites (Figure 3A). The changes in poly(A) site use suggest a primarily repressive function of MATR3/PTBP1 binding (Figure 3D). In the most extreme cases, recognition of poly(A) sites within LINEs results in complete loss of all downstream exons upon combined depletion of MATR3 and PTBP1 (i.e., in *MROH1* and *PIGN1*) (Figures S4A and S4B). Metaprofiles of iCLIP data confirmed the direct binding of MATR3 and PTBP1 to repressed poly(A) sites (Figure 3E). Thus, we conclude that MATR3 and PTBP1 are potent repressors of RNA processing at LINEs, preventing the use both of poly(A) sites and splice sites.

### Deletion of an Intronic LINE Disrupts MATR3-Dependent Repression of a Cryptic Exon in *ACAD9*

**Figure 4:**
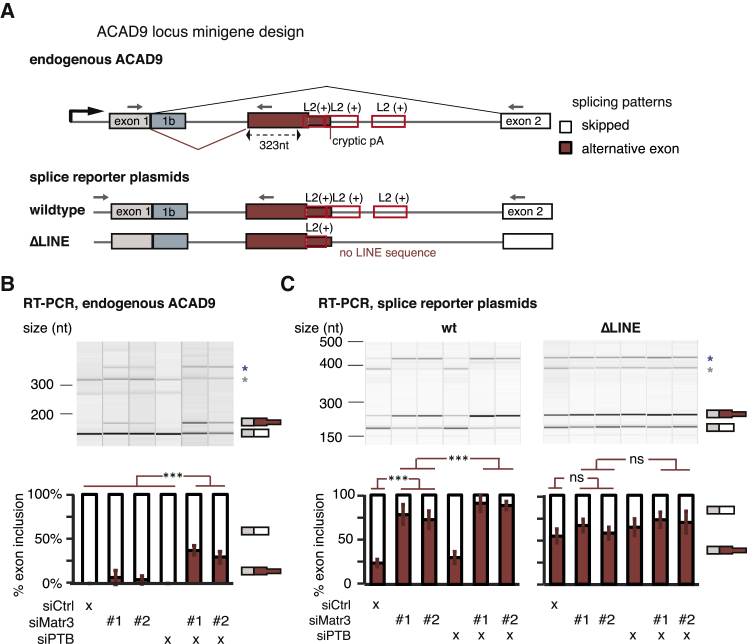
Partial Deletion of L2 Sequences Disrupts Splicing Repression of *ACAD9* by MATR3/PTBP1 (A) Schematic illustrating the endogenous ACAD9 locus and the ACAD9 splice reporter. The first two exons and the complete intron1 were cloned into a CMV-driven reporter plasmid. In the ΔLINE splice reporter, 499 bp of L2 sequence were replaced by non-repetitive sequence of intron2 of ACAD9. Arrows indicate positions of primers used for isoform detection in RT-PCR. (B) The inclusion level of the LINE-proximal alternative exon in endogenous ACAD9 was measured in total RNA of cells depleted of MATR3 and PTBP1/2 individually or in combination. (C) The inclusion level of the LINE-derived exon was measured as in (B) in the wild-type and ΔLINE ACAD9 splice reporter. (B and C) To test for significance, one-way ANOVA was used coupled with multiple comparison correction according to Tukey’s HSD. ^∗∗∗^p value below 0.001. Semiquantitative RT-PCR analysis is averaged across three independent replicates, error bars indicate SD. Additional splice products are indicated by asterisks; these include a longer form of exon1 with an alternative 5′ splice site (*exon 1b*). For simplicity, only the relevant isoforms are quantified. See also Figure S5.

**Figure S5:**
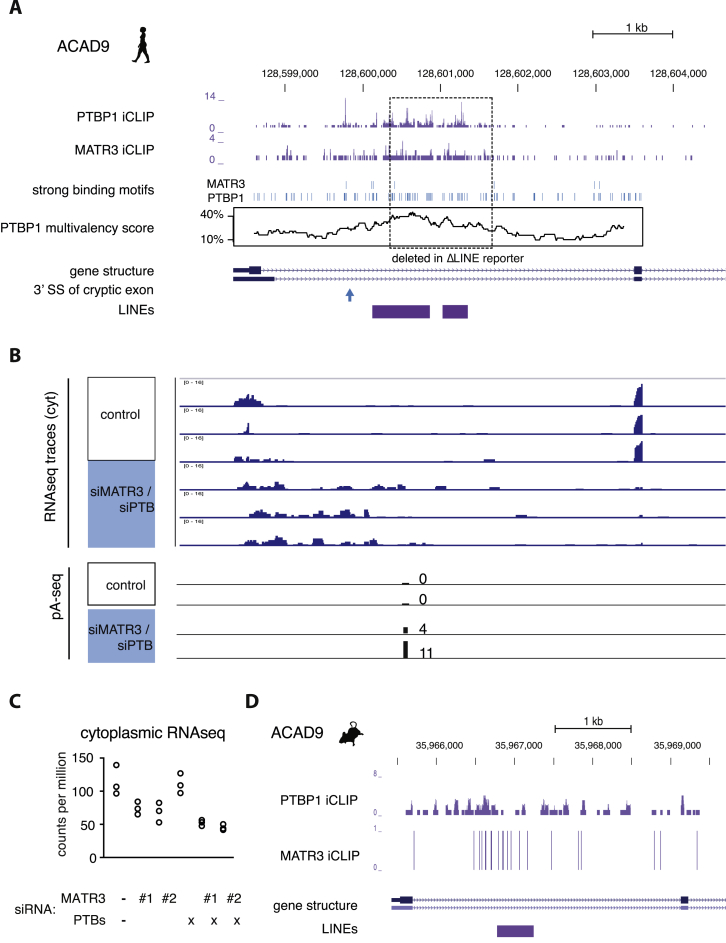
Depletion of *ACAD9* Expression following Inclusion of a LINE-Derived Exons, Related to Figure 4 (A) Genome browser tracks for PTBP1 and MATR3 iCLIP data from HeLa cells at the *ACAD9* locus relative to binding motifs of PTBP1 and MATR3. Multivalency of PTBP1 binding sites is indicated as percent of nucleotides that are part of a binding motif within 250 nucleotide windows. Below, the structure of annotated *ACAD9* transcripts is annotated as well as the position of the 3′ splice site of the cryptic exon repressed by MATR3/PTBP1 and the position of L2 element fragments. (B) Stranded RNaseq data from cytoplasmic RNA of HeLa cells depleted of MATR3 and PTBP1/2 is shown. Below the position of a new pA site within the second L2 repeat is shown, which is only detected in absence of MATR3/PTBP1/2. (C) Quantification of *ACAD9* expression in single and combined depletion of MATR3 and PTBP1/2 from cytoplasmic RNaseq. (D) Genome browser tracks for PTBP2 and MATR3 on the mouse *Acad9* locus. In mouse, there is a single, 465bp long L2 insertion annotated.

We chose to examine in detail repression of a cryptic exon in intron1 of *ACAD9* by MATR3 and PTBP1. Intron1 of *ACAD9* contains three fragments of L2 elements in sense orientation (Figure 4A) with multivalent PTBP1 binding sites, which are strongly bound by MATR3 and PTBP1 in cultured human cells as well as in mouse brain (Figures S5A and S5D). We confirmed by RT-PCR and Sanger sequencing that depletion of MATR3 led to the inclusion of an alternative exon with a 3′ splice site that is located 323 nt upstream of the nearest L2 repeat. Even though depletion of PTBP1/2 on its own did not affect the exon, its inclusion was more pronounced after co-depletion of MATR3 and PTBP1/2 compared to depletion of MATR3 alone (Figure 4B). 3′ end sequencing data showed the emergence of a cryptic poly(A)-site within the L2 sequences that is only used in MATR3 and PTBP1/2 depleted cells, suggesting the exon is an alternative terminal exon. Moreover, expression of *ACAD9* gene was 2-fold decreased upon depletion of MATR3 and 3-fold decreased upon combined depletion of MATR3 and PTBP1/2 (Figures S5B and S5C). To confirm that MATR3 and PTPB1 repress the exon by binding to the downstream L2 elements, we designed a *ACAD9* splicing reporter plasmid comprising exon1, the complete intronic sequence including all three L2 repeats (wild-type) and exon2, and a mutant reporter that lacked two L2 repeats and the multivalent PTBP1 binding sites within them (ΔLINE). The wild-type reporter reproduced the splicing pattern of the endogenous sequence before and after depletion of MATR3 and PTBP1 (Figure 4C), albeit with a generally more prominent inclusion of the LINE-proximal exon. Importantly, inclusion of the LINE-proximal exon strongly increased in the ΔLINE reporter, with loss of regulation by MATR3/PTBP1 (Figure 4C). Hence, the L2 sequence downstream of the exon appears essential for the capacity of MATR3/PTBP1 to repress the exon. We conclude that MATR3 and PTBP1 directly bind LINEs to synergistically repress the use of splice and poly(A) sites within and close to the intronic LINEs in *ACAD9*.

### Evolutionarily Old LINEs Are a Major Source of Mammalian Alternative Exons

**Figure S6:**
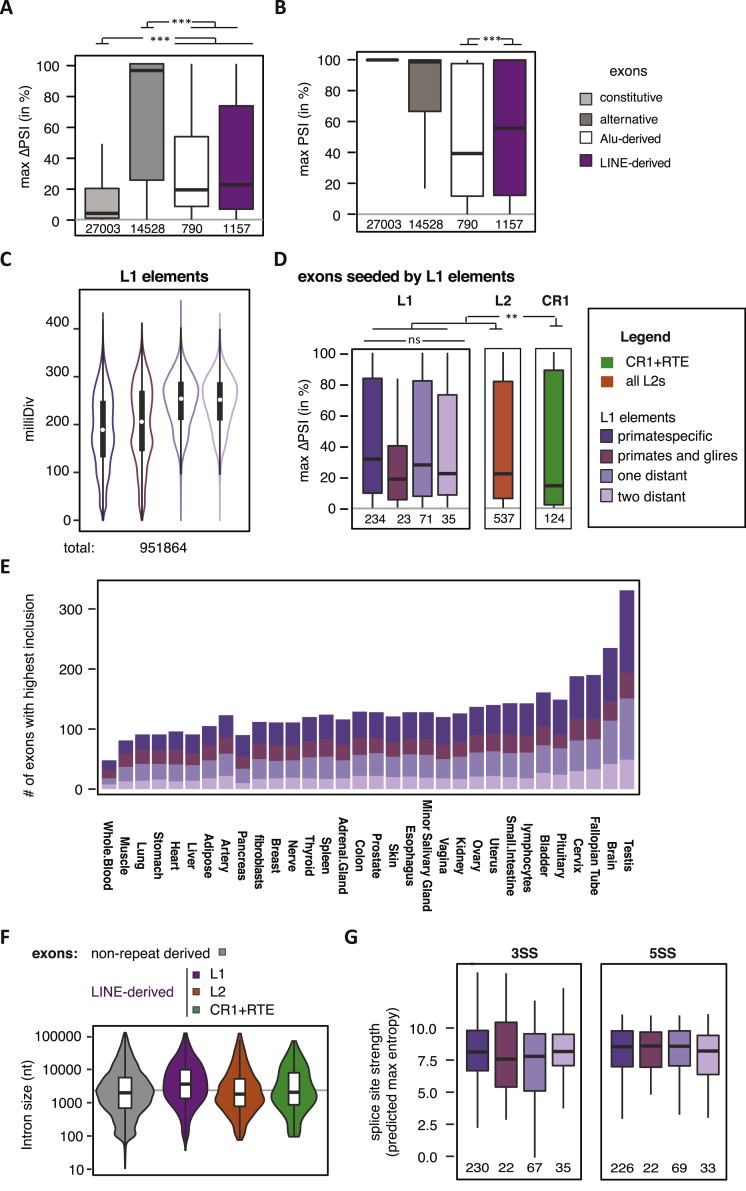
L1-Derived Exons Are a Source of Primate-Specific Alternative Exons with High Tissue Specificity, Related to Figure 5 Percent splice index (PSI) was calculated in the GTEx panel of human tissues for LINE-derived and Alu-derived exons, as well as all other exons of the same genes. All exons are annotated within UCSC and cross-referenced with RefSeq annotation. Inclusion levels range from 0 to 100%, showing no inclusion or full inclusion. If no support for expression of the flanking exons was found, the gene is assumed to be non-expressed. The number of exons in each group is indicated at the bottom of each boxplot. Genomic age of L1 elements as defined and color-coded in Figure 5A. Significance tests were done across groups by Kruskal-Wallis’ test and pairwise comparisons were corrected according to Siegel-Castellan. ^∗∗^ and ^∗∗∗^ indicate adjusted p value was below 0.01 and 0.001, respectively. (C-E, G): Groups are color coded as indicated in the legend on the right of panel D. (A) For all exons surveyed within the GTEx data, the difference in PSI between the tissues with highest and lowest inclusion was calculated as metric for tissue-specific inclusion. (B) For all exons surveyed within the GTEx data, the difference in PSI between the tissues with highest and lowest inclusion was calculated as metric for tissue-specific inclusion. (C) The substitutions from L1 consensus families is shown for L1s grouped by phylogenetic age. As expected, young elements show fewer substitutions from consensus then old elements. (D) Difference in PSI between tissues with highest and lowest inclusion for exons derived from L1 elements grouped by genomic age of the insertion, compared to exons derived from L2 and CR1 insertions. (E) The number of L1-derived exons is shown for all primary tissues screened in the GTEx data, based on testing in which tissue an exon is most included. Exons are allowed to be counted multiple times if maximum inclusion was in multiple tissues, for instance because they are constitutive. (F) UCSC annotated L1-derived exons are within long introns. Intron size is the total distance between the flanking exons. The gray line indicates an intron length of 2kb. (G) Exons derived from L1 elements have strong splice sites irrespective of the genomic age of the insertion. The maximum entropy score of 5′ and 3′ splice sites of each exon was predicted based on nucleotide sequence (Yeo and Burge, 2004).

**Figure 5:**
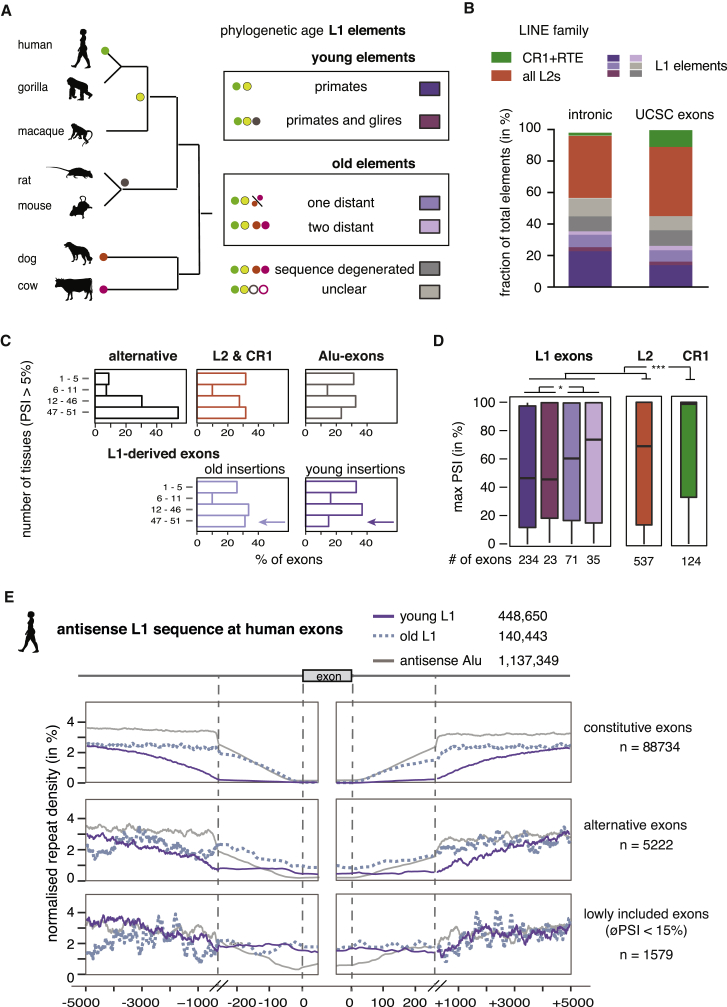
Evolutionarily Old LINEs Are a Source of Lineage-Specific Alternative Exons (A) The phylogenetic age of each LINE fragment in the human genome was mapped by comparison to the gorilla, rhesus macaque, mouse, rat, dog, and cow genome assemblies using UCSC liftover genome alignments overlaid with RepeatMasker annotation. Elements specific to the primate or euarchontoglires lineage are considered evolutionarily young elements, while elements present in cow and dog are considered old elements. Phylogenetic groups are color-coded and used in analysis (B–E). (B) Percentage of UCSC annotated exons derived from phylogenetic groups as defined in (A). Exons are generally not derived from the youngest L1 elements. (C) Exons derived from evolutionarily young L1 elements are rarely used across many tissue subtypes in human. Percent spliced index (PSI) was calculated in the GTEx panel of human tissue samples for LINE-derived exons annotated in UCSC. We determined the number of tissues in which each exon was detectable at PSI >5% and compared repeat-derived exons to non-repeat derived alternative exons. (D) Maximum inclusion in any tissue correlates with the genomic age of L1-derived exons. Significance was tested across groups by Kruskal-Wallis’ rank-sum test. The number of exons in each group is indicated at the bottom; ^∗^adjusted p values below 0.05, ^∗∗∗^adjusted p values below 0.001. (E) Density profiles showing L1 antisense sequence 5 kb upstream and downstream of human exons. L1s were split for evolutionary young and old insertions and repeat density is normalized to the total number of repeats in the two groups. For comparison, the primate-specific Alu insertions are shown. Exons were grouped by inclusion in human tissues into those that are >5% but on average <15% included in any tissue, those which are alternative, and those which are constitutively included. To better present the repeat density around the splice sites, the x axis is cut at 250 nt to show a zoom-in of the 250 nt flanking the exons. øPSI, average PSI across 51 tissues. See also Figure S6 and Tables S5 and S6.

To assess the impact of LINE-derived exons on transcriptomes of human tissues, we used the RNA-seq data available from the GTEx Consortium (2015) (V6p data). We monitored inclusion of 45,940 exons of 4,566 genes in RNA-seq data across 51 tissues, including all known LINE-derived exons. 1,154 LINE-derived exons had 5% inclusion in at least one tissue; in contrast to other alternative exons, LINE-derived exons are rarely switch-like events (Figure S6A) but are generally more highly included than the primate-specific Alu-derived exons (Figure S6B), which suggests a correlation between evolutionary age and formation of new exons at repetitive elements. To study this further, we estimated the evolutionary age of individual L1 elements. We performed cross-species comparison of all human L1 elements with two primate genomes, two rodents, and one each of the carnivore and laurasiatherian lineages (Figure 5A) and annotated the age of all L1 elements according to their most likely time of insertion as primate-specific (459,702), euarchontoglires-specific (38,642), or as more ancient elements that inserted before the mammalian radiation (142,739). We further categorized mammal-wide insertions by assigning if they were present in dog and cow (two distant species) or only in one of them (one distant), which might indicate differences in selective pressure for their retention. The divergence from the consensus of the corresponding L1 family confirmed our age estimates determined by cross-species comparison (Figure S6C).

Notably, the exons derived from primate-specific L1 elements were highly tissue-specific, because they were rarely present in all of the 51 tissues and had the highest difference in inclusion between any pair of tissues (Figures 5C and S6D). Conversely, the highest inclusion in any tissue of exons from primate-specific L1 elements was lower than for exons derived from evolutionarily older L1 elements and lower than for exons derived from L2 and CR1 elements (Figure 5D). Between tissues, we found highest inclusion of LINE-derived exons was often in tissues of the reproductive system and the brain (Figure S6E).

CR1 and L2 elements are less prevalent in human, but are substantially older than L1 elements, because most inserted before the mammalian radiation (Deininger and Batzer, 2002). We identified 594 L2- and 150 CR1-derived exons with >5% inclusion in at least one tissue. Exons derived from L2 elements have similar inclusion levels to the well-preserved mammal-wide L1 insertions, and CR1-derived exons have the highest inclusion levels (Figure 5D). Taken together, we show that the use and inclusion level across tissues of LINE-derived exons increases with the evolutionary age of the LINE.

### Evolutionarily Young LINEs Are Generally Confined to Deep Intronic Regions

**Figure S7:**
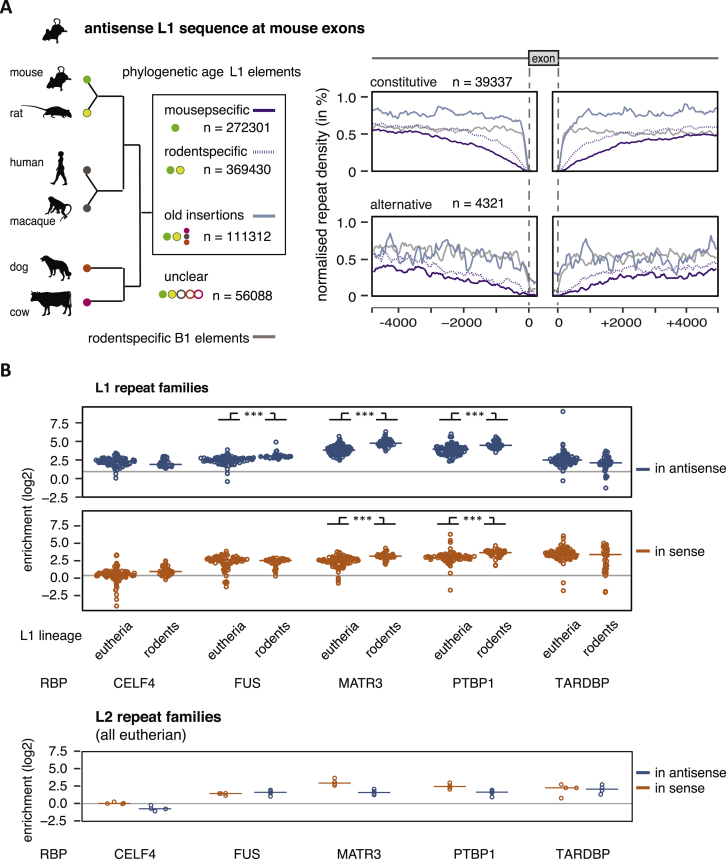
Murine MATR3 and PTBP1 Bind to Mouse-Specific L1 Insertions, Related to Figure 6 (A) Density profiles showing L1 antisense sequence 5kb upstream and downstream of constitutive and alternative exons in the mouse. The genomic age of each L1 element in the mouse genome was mapped by comparison to the rat, rhesus macaque, human, dog and cow genome assemblies. For comparison, the rodent-specific B2 repeat insertions are shown. (B) TEtranscript (Jin et al., 2015) was used to estimate the enrichment of each subfamily of L1 and L2 repeats among the bound RNA sequences of a panel of RBPs, with CLIP data available for C57Bl mouse brain; comparing the abundance in recovered eCLIP tags to the abundance in RNaseq reads of ENCODE sequencing data of mice at P2. For each RBP, 133 repBase LINE subfamilies were considered (129 for L1, 4 for L2) (Jurka, 1998). Families were grouped depending on if they emerged in eutheria or only in rodents, based on the information available on repBase. Since eCLIP is strand-specific, binding to LINEs transcribed in sense or in antisense was quantified separately, colored in red and blue. Details and references of datasets are given in Table S1. Differences between rodent-specific and mammalian/eutherian L1 families were tested by two-sided t test and corrected for multiple testing according to Bonferroni.

MATR3 and PTBP1/2 preferentially repress LINE-derived exons that are located within long introns (Figure S3G). Notably, among all LINE-derived exons, only exons from L1 elements were located within particularly long introns (Figure S6F). To better understand how phylogenetic classes of L1s are positioned in pre-mRNAs, we examined their distribution around different types of exons. Strikingly, we found that young, antisense L1 elements were almost completely depleted from the 500-nt regions around constitutive exons and less frequent up to 3 kb away from exons compared to old LINEs (Figure 5E). Older L1s were well tolerated up to 250 nt at all exons, and their depletion is apparent only in close vicinity of constitutive exons. A milder exclusion of young L1s was seen around alternative exons, with no exclusion around exons with low inclusion across human tissues (average percent spliced index [PSI] <15%), indicating that the L1 might contribute to the repression of these exons. In contrast to the primate-specific antisense L1s, the primate-specific Alu repeats were only excluded from the immediate vicinity of exons, but not from the flanking intronic regions. To assess if selection pressure against young L1s takes place also in other species, we classified the evolutionary age of mouse L1 elements and repeated the analysis on mouse L1-derived exons. Consistently, mouse- and rodent-specific L1 were excluded from the vicinity of constitutive exons in a similar pattern as the primate-specific L1s in human (Figure S7A). Just like Alu repeats in human, the rodent-specific B1 elements were only excluded from the immediate vicinity of exons. Overall, this indicates that evolutionarily young, antisense L1 elements are under particularly strong negative selection in the vicinity of established exons, both in primates and in rodents.

### Phylogenetic Groups of LINEs Differ in Their RBP Interactome

**Figure 6:**
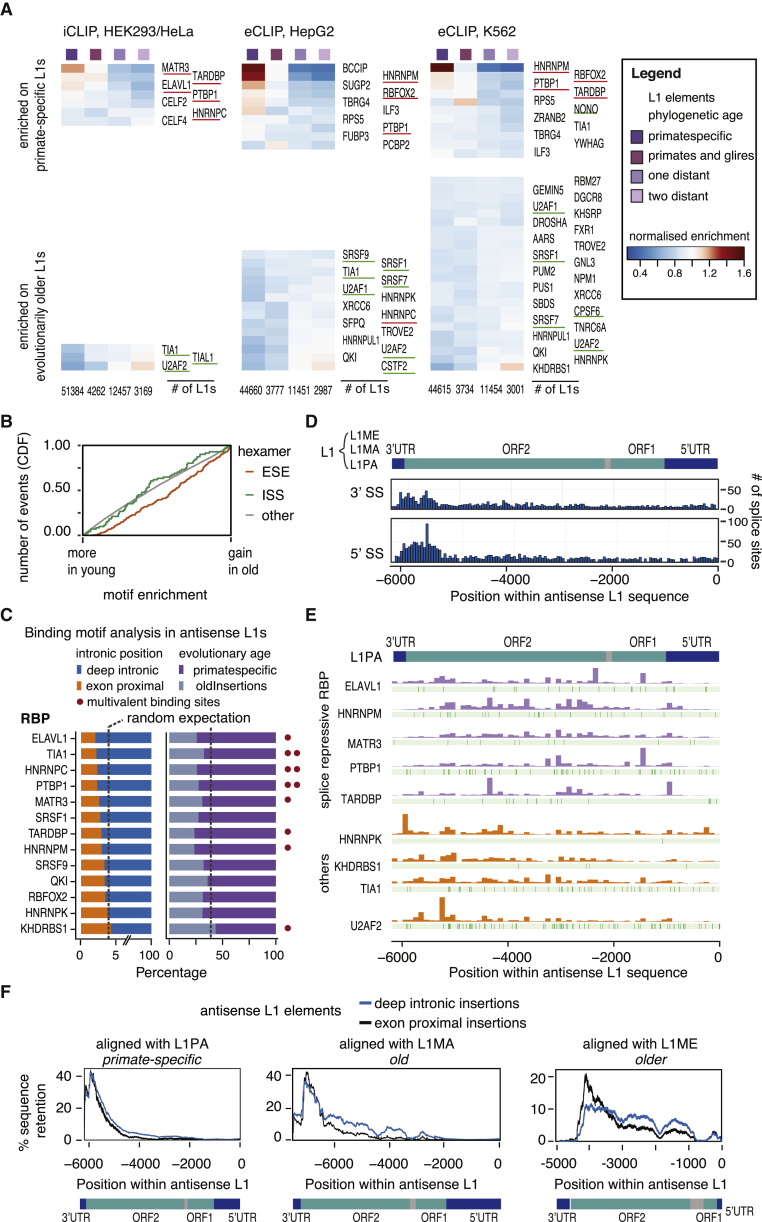
Young L1 Elements Are Rich in Splice Repressor Binding Motifs that Are Lost in Evolutionarily Older Element (A) RBPs show preferences for binding to L1 elements of different evolutionary ages. The L1 elements with 10% highest coverage across any i/eCLIP data were used to calculate a relative binding estimate for each RBP ranging from 0 to 1, and for visualization of binding preference, the enrichment of each RBP was normalized to its mean. The number of L1 elements considered in each cell line is given at the bottom. RBPs considered splice-repressive are underlined in red, and components of the RNA processing machineries in green. (B) Cumulative distribution function of gain or loss of exonic splice enhancer (ESS) and intronic splice silencers sequences (ISS). All hexamer sequences were ranked by their enrichment in evolutionarily young compared to old LINEs. (C) Antisense L1 sequences with known binding motifs for relevant RBPs, and the percentage of evolutionarily young versus old elements among them and the percent of deep intronic versus exon-proximal elements. The dotted line indicates the expected proportion. RBPs with multivalent binding sites are marked with one or two red dots, if 10% and 20% of the 100-nt window were part of the motif, respectively. We used the top 10% of L1 sequences with the highest density of binding motifs within a 100-nt window. (D) The position of splice sites of L1-derived exons across the L1 sequence. For reference, the structure of the L1PA family of L1s is given on top. Only splice sites in antisense L1 elements are shown. (E) The position of RBP binding motifs within the antisense L1PA family consensus sequence in green. On top of the track with each RBP’s binding motifs, coverage in e/iCLIP binding data is shown. (F) Alignment of antisense L1 insertions against L1 consensus sequences. We selected deep intronic insertions (shown in blue) and exon-proximal insertions (in orange) and aligned them against three consensus families, only keeping the best alignment for each genomic insertion. See also Figure S7 and Table S7.

In spite of their distinct inclusion levels, we did not find any marked differences in the splice site strengths of LINE-derived exons derived from elements of different of the phylogenetic age (Figure S6G). Therefore, we reasoned that differential binding of regulatory RBPs might determine the exonization of LINEs. To test this hypothesis, we exploited the available iCLIP and eCLIP data to analyze RBP binding profiles. In total, 126,628 LINEs contained one cDNA per million read for at least one of the 49 LINE-binding RBPs in at least one human cell line, including 93,420 L1 elements. We calculated a relative binding score for each RBP relative to the average binding of all 49 RBPs on each L1 and visualized binding preferences across the phylogenetic groups of L1 elements. Strikingly, MATR3 was the RBP with strongest iCLIP enrichment on primate-specific L1s, and PTBP1 was enriched on these both in iCLIP and eCLIP (Figure 6A). Similarly, we find that MATR3 and PTBP1 preferentially bind to L1 families that are evolutionarily young in the mouse (Figure S7A). Given our finding that MATR3 and PTBP1 can inhibit splicing and 3′ end processing in the vicinity of LINEs (Figure 3A), their preferential binding to evolutionarily young LINEs could contribute to the negative selection against presence of these LINEs close to exons (Figure 5E).

Notably, most RBPs enriched on primate-specific L1s are known splicing repressors (underlined in red in Figure 6A) (Coelho et al., 2015, Ling et al., 2015, Xu et al., 2014, Lebedeva et al., 2011, Damianov et al., 2016). In contrast, RBPs enriched on evolutionarily older L1s include many factors known to enhance splicing or 3′ processing, including SR proteins, U2AF2, CSTF2, and CPSF6 (underlined in green in Figure 6A). Thus, decreased binding of repressive RBPs, accompanied by binding of splice-promoting RBPs, could explain why the evolutionarily younger L1s are mainly restricted to deep intronic regions, while older L1s are the more common source of exons.

### High Density of RBP Binding Motifs within L1 Recruits RBPs to Repress Cryptic Splice Sites

Given the selection against antisense L1 sequences proximal to exons, and their RBP interactome, we predicted that antisense L1 sequence contain splice-repressive sequences. As a first step, we examined the presence of exonic splice enhancer (ESEs) and intronic splice silencer sequences (ISSs), as defined by previous studies (Fairbrother et al., 2002, Wang et al., 2013). This showed that the ratio of ESEs to ISS increases with the age of L1 elements (Figure 6B). Next, we searched for motifs recognized by the RBP preferentially binding to antisense L1 elements (Table S7, motifs and references in Table S1). Among the pentamers that change most in frequency from evolutionarily young to old insertions, we found several binding motifs recognized by the LINE-binding RBPs, particularly, evolutionarily older L1s contained fewer binding motifs of ELAVL1, PTBP1, HNRNPC, and HNRNPM, but increased incidence of multiple binding motifs for KHDRBS1 (false discovery rate [FDR] <0.05). We noticed that often L1 elements contained many copies of a particular splice-repressive motif, while other L1 elements would be entirely devoid of it. Therefore, we also counted the highest density of individual motifs within 100 nucleotides. Particularly common were repeats of binding motifs for HNRNPC, PTBP1, ELAVL1, TIA1, TADRBP, and HNRNPM. The L1 elements with such repeats were more often primate-specific and deep-intronic than expected by chance (Figure 6C).

Putative splice sites are common across the L1 sequence (Belancio et al., 2006). To understand better their positions at LINE-derived exons, we plotted splice sites of these exons along the consensus sequence model of L1 and L2. Notably, most splice sites are derived from a hotspot at the boundary between ORF2 and the 3′ UTR region of L1 and L2 (Figure 6D). In the consensus sequence, binding motifs of a number of splice repressors were common across the region encoding for L1 ORF2p and often repeated within a few 100 nt (Figure 6D). In contrast, binding motifs of repressive RBPs such as PTBP1 are common across the body of a full-length L1 element, particularly in the region encoding the L1 ORF2p, where they can form multivalent binding sites, some of which span over 100 nt (Figure 6E). To understand how such motifs influence RBP binding patterns, we mapped the e/iCLIP data of the most relevant RBPs onto the consensus sequence of the primate-specific L1 family, L1PA. We found that many repressive RBPs primarily bind within the region encoding ORF1p and ORF2p, including ELAVL1, MATR3, PTBP1, TARDBP, and HNRNPM, while the region close to the 3′ UTR commonly gives rise to exons.

Finally, we asked if the binding sites for repressive RBPs are part of the regions that are selected against within exon-proximal L1 elements. We aligned groups of exon-proximal and deep-intronic antisense L1s against the consensus sequences of the most common L1 families (Figure 6F). All three families showed strong 5′ truncation, leading to loss of ORF1p and a major portion of ORF2p sequence, but the extent of deletion of the ORF2p region was stronger at exon-proximal elements in all families. The difference between deep intronic and exon-proximal L1s was clearest for the mammal-wide families, L1MA and L1ME, which are old enough to have undergone significant divergence upon selective pressure. We conclude large parts of the ORF2p region are widely selected against in exon-proximal insertions, which coincides with the regions containing the largest density of binding motifs for repressive RBPs. The binding sites for repressive RBPs are a likely reason for negative selection of young L1 elements from the proximity to exons. We hypothesize that removal of these repressive sites upon evolutionary divergence of antisense L1s decreases the negative selection, allowing them to be located closer to exons, and to seed L1-derived exons.

## Discussion

**Figure 7:**
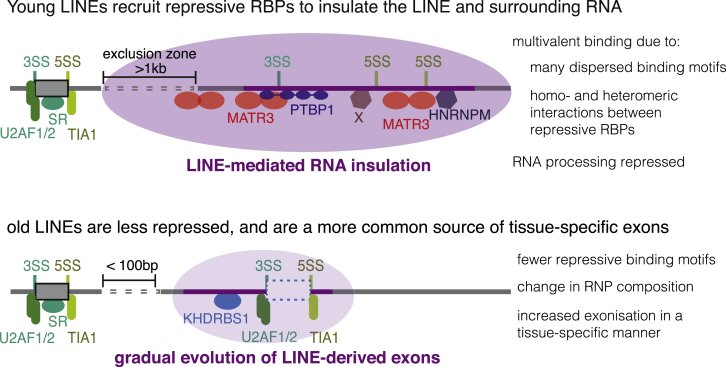
Evolution of LINEs from RNA Insulation to a Template for New Exons Consensus L1 elements contain strong putative splice sites, but exonization is rare. Evolutionarily young L1s recruit a number of splice repressive proteins, including MATR3, PTBP1, and HNRNPM, as well as RBPs of yet unknown function (indicated by X; including BCCIP and SUGP2, see Figure 6A). These proteins recognize RNA motifs present within the L1 elements. The extent of splice-repressive proteins assembling on the L1 elements leads to selective pressure against young L1 insertions in a large proximity window of established exons. Evolutionarily older elements have a high probability of losing binding sites of repressive RBPs. Their exonization is more common, but still largely tissue-specific.

We analyzed the binding patterns of dozens of RBPs across multiple cell lines to find that the RBPs assemble on over 100,000 LINEs in transcripts of human genes. The most common of these are the antisense L1 elements. The evolutionarily young L1s primarily recruit RBPs that repress RNA processing, and thereby they insulate the deep intronic RNA from the splicing and polyadenylation machineries (Figure 7). These young L1 elements are depleted from exon proximal regions, indicating that they are under negative selection due to their repressive effects on adjacent exons. We found a high density of binding motifs for repressive RBPs and of intronic splice silencer sequences in young antisense L1s, providing the potential for multivalent RBP:RNA binding. Accumulation of mutations in evolutionarily older LINEs decreases the number and multivalency of these splice-repressive motifs. This leads to decreased binding of repressive RBPs, allows older LINEs to be positioned closer to exons, and makes them accessible to the splicing machinery. As a result, older LINEs more often give rise to alternative exons that are highly included in transcripts expressed across many human tissues.

### The Potential for Oligomeric RNP Assembly on LINEs by Multivalent Binding Sites

We describe more than 20 RBPs with enriched binding to LINEs, and we show that antisense L1 elements, in particular, tend to contain a high density of binding motifs for many of these RBPs. It is likely some of these RBPs bind LINEs co-operatively and as part of larger RBP complexes. We directly demonstrate that MATR3 is required for efficient crosslinking of PTBP1 to antisense L1 elements, which contain multivalent binding sites. This can be modeled in *in vitro* experiments, and we show that a linear peptide motif in MATR3 is strictly required for the two proteins to simultaneously bind to RNA *in vitro*. We expect that many more RBP:RBP interactions contribute to efficient recruitment of splicing repressors to LINEs. MATR3 has been reported as part of several nuclear multimeric complexes (Damianov et al., 2016, Zhang and Carmichael, 2001, Iradi et al., 2018) that include HNRNPM, RBFOX1 and ILF3, all three of which we found to be also enriched at antisense L1 elements. It remains to be seen if MATR3 contributes to the enriched binding of these RBPs to the young antisense L1s.

In light of the recent observations that multivalent interactions are required for phase-transitioning of several RBPs (Lin et al., 2015, Banani et al., 2017), the high density of binding sites within antisense L1s is striking. Clustered RNA binding motifs can enable RBPs to achieve high-affinity binding by cooperative interactions with multiple RNA binding domains, as for instance shown for PTBP1 (Cereda et al., 2014). PTBP1 and other RBPs can form oligomers and undergo liquid phase separation when bound to RNA (Banani et al., 2017). Antisense L1 elements might be a suitable scaffold for formation of heteromeric complexes through multivalent binding. In case of MATR3 and PTBP1, binding of both proteins is clearly necessary for and more effective in repression of LINE-derived exons. It will be of prime interest to understand further if interaction surfaces that promote phase separation are necessary to effectively insulate repetitive elements from the processing machineries.

Mutations in the intrinsically disordered regions of a number of RBPs, including MATR3, can lead to neuronal death in amyotrophic lateral sclerosis (Bakkar et al., 2018), and these mutations often change the dynamics of RNP assembly. Deregulation of retrotransposons, including LINEs, has been observed in a *Drosophila* model of ALS (Krug et al., 2017), therefore we speculate that mutation-driven changes in the RNP assembly on LINEs could also contribute to neuronal diseases in humans.

### Could the Repressive Effects of Young LINEs Explain Their Exclusion Zone around Exons?

The decreased abundance of LINEs in the immediate vicinity of splice sites has been previously observed (Zhang et al., 2011, Corvelo and Eyras, 2008), and we now find that this depletion is dependent on their evolutionary age, with a strongest depletion seen for evolutionarily young LINEs. Multiple scenarios could result in purifying selection against exon-proximal young LINEs in a species, and they are not mutually exclusive: (1) purifying selection against LINEs when inserted close to exons, due to their inherent capacity to repress splicing of nearby exons; (2) *de novo* formation of exons only outside the repressive environment created by LINEs; and (3) the accumulation of sequence truncations and mutations in LINEs that decrease their repressive capacity. The prevalence of antisense L1 elements in deep intronic regions is likely a major factor for the accumulation of splice silencer sequences in human introns. LINEs also allow accumulation of cryptic splice sites within large introns, as a consequence of the insulation by repressive RBPs. It is possible that additional pathways contribute to this insulation, such as the low number of splice enhancer sequences, DNA methylation and repressive chromatin.

LINEs are the most prevalent repetitive elements in the human genome thus greatly contributing to the increase of intron size in mammals. It is striking that the consensus sequences of antisense young L1 families are rich in intronic splice silencer sequences and binding motifs of splice-repressive RBPs, and the antisense orientation is twice as common in introns. This suggests that new insertions are immediately repressed when in antisense orientation, which likely allows them to persist and contribute to the expansion of mammalian introns throughout evolution.

### LINEs Facilitate the Evolution of RNA Processing

The highly multivalent sites bound by repressive RBPs are often lost in the older L1s due to their more diverse sequences. Removal of those repressive binding sites is paralleled by a relative increase in binding of splice-promoting RBPs and facilitates evolution of new exons. The relationship between repressive RBPs and LINEs is in many ways similar to the evolutionary dynamics of KAP1/KRAB transcription factors, which repress transcription preferentially at young retrotransposons, and confer robustness to transcriptional networks while facilitating evolutionary innovation (Castro-Diaz et al., 2014, Thomas and Schneider, 2011, Imbeault et al., 2017).

We observe that L1-derived exons are highly tissue-specific, and the highest number of them is found in the testis and the brain (Figure S6E). The testis is known to be promiscuous in its transcriptional output, which has been suggested to facilitate gene birth (Kaessmann, 2010). Similarly, varying activity of repressive RBPs across tissues might facilitate the creation of new LINE-derived exons in specific cell types. Moreover, mutations in RBPs or in LINEs themselves could cause disease through aberrant splicing of LINE-derived exons.

## STAR★Methods

### Key Resources Table

**Table:** 

REAGENT or RESOURCE	SOURCE	IDENTIFIER
**Antibodies**		

Rabbit anti PTBP1/2, serum	C. Smith; Spellman et al., 2007	N/A
Rabbit polyclonal anti MATR3	Insight	GTX47279; RRID: AB_11170111
	Biotechnology	
Rabbit anti GAPDH	Cell Signaling	14C10; RRID: AB_10693448

**Chemicals, Peptides, and Recombinant Proteins**		

4-thiouridine	Sigma	Cat# T4509-100MG
Blue Sepharose 6 Fast Flow/ HisTrap HP columns	GE LifeSciences	Cat# 17-0412-01
RevertAid enzyme	Fermentas	Cat# 10387979
Trizol LS	Life Technologies	Cat# 10296028
Zymo Direct-zol	Zymogen	Cat# R2052
RNA MiniPrep columns		

**Critical Commercial Assays**		

RiboZero	Epicenter	Cat# MRZG12324
TruSeq stranded total RNA Sample Prep Kit	Illumina	Cat# 20020599
QuantSeq mRNA 3′ end sequencing kit	Lexogen	Cat# SKU 015.96 and SKU 016.96

**Deposited Data**		

3′ end profiling of HeLa cells depleted of MATR3, PTBP1/2	This paper	E-MTAB-6287. Accessible via https://www.ebi.ac.uk/arrayexpress/
RNA-seq of HeLa cells depleted of MATR3, PTBP1/2	This paper	E-MTAB-6204. Accessible via https://www.ebi.ac.uk/arrayexpress/
iCLIP of MATR3 from C57BL/6J wildtype mice	This paper	E-MTAB-6283. Accessible via https://www.ebi.ac.uk/arrayexpress/
4SU-iCLIP of MATR3 from HEK293 cells with or without PTBP1 depletion	This paper	E-MTAB-6267. Accessible via https://www.ebi.ac.uk/arrayexpress/
4SU-iCLIP of PTBP1 from HEK293 cells with or without MATR3 depletion	This paper	E-MTAB-6286. Accessible via https://www.ebi.ac.uk/arrayexpress/
22 further iCLIP datasets for different RBPs	This paper	Datasets used are listed in Table S1. Accessible via https://imaps.genialis.com/
All eCLIP data	ENCODE Consortium	Datasets used are listed in Table S1. Accessible via https://www.encodeproject.org/search/?type=Experiment&assay_title=eCLIP
RNaseq data of HepG2 and K562 cells	ENCODE Consortium	ENCSR885DVH; ENCSR181ZG; GSE90238; GSE90256; GSE90249; GSE90230; GSE90220; GSE90248; GSE90250; GSE90228; GSE90236; also listed in Table S1.
Analyzed data, Table S5. Phylogenetic age of L1 elements	This paper	https://data.mendeley.com/datasets/56sxpgs4d9/1
Analyzed data, Table S6. Inclusion of 43583 human exons in GTEx V6p consortium data.	This paper	https://data.mendeley.com/datasets/s9d9nsysjz/1
Human reference genome UCSC assembly hg19 (GRCh37)	Genome Reference Consortium	http://hgdownload.cse.ucsc.edu/goldenPath/hg19/
Repeat Masker genome annotation	Smit et al., 1996–2004	RRID: SCR_012954
RepBase	Jurka, 1998	N/A

**Experimental Models: Cell Lines**		

HEK293T	ATCC: CRL-3216	RRID: CVCL_0063
HeLa	ATCC: CCL-2	RRID: CVCL_0045

**Experimental Models: Organisms/Strains**		

Mouse: C57BL/6J	Laboratory for Molecular Biology, Cambridge	N/A

**Oligonucleotides**		

MATR3 siRNA (#1)	Invitrogen	HSS114732
MATR3 siRNA (#2)	Invitrogen	HSS114730
PTBP1 siRNA, AACUUCCAUCAUUCCAGAGAA	Dharmacon	Customized product
PTBP2 siRNA, AAGAGAGGAUCUGACGAACUA	Dharmacon	Customized product
control siRNA	Invitrogen	Cat. #12935-300
RNA oligonucleotides with AUCUU and CTCTT binding motifs; see Methods for full sequences	SIGMA DNA oligonucleotides cloned into pGEM4Z	Customized product

**Recombinant DNA**		

pGEM4Z	Promega	pGEM4Z

**Software and Algorithms**		

Bowtie2	Langmead and Salzberg, 2012	http://bowtie-bio.sourceforge.net/bowtie2/index.shtml
TopHat2	Kim et al., 2013	RRID: SCR_013035; http://tophat.cbcb.umd.edu/
STAR	Dobin et al., 2013	RRID: SCR_015899; https://github.com/alexdobin/STAR
Cufflinks	Trapnell et al., 2012	RRID: SCR_014597; http://cole-trapnell-lab.github.io/cufflinks
expressRNA	Rot et al., 2017	http://www.expressrna.org/
Whippet	Sterne-Weiler et al., 2017	https://github.com/timbitz/Whippet.jl
BLAST+/2.3.0	Camacho et al., 2009	RRID: SCR_001598; https://blast.ncbi.nlm.nih.gov/Blast.cgi
R	R Project for Statistical Computing	RRID: SCR_001905; http://www.r-project.org/

### Contact for Reagent and Resource Sharing

Further information and requests for resources and reagents should be directed to and will be fulfilled by the Lead Contact, Jernej Ule (jernej.ule@crick.ac.uk).

### Experimental Model and Subject Details

#### Cell lines

HEK293T and HeLa cells were purchased from ATCC (CRL-3216 and CCL-2; both of female origin). Both cell lines were maintained in DMEM with 10% FBS at 37°C with 5% CO2 injection, and routinely passaged twice a week. Cell lines were confirmed to be mycoplasma-free with repeated testing, using either the LookOut Mycoplasma PCR Detection Kit or the MycoAlert mycoplasma detection kit (Lonza). Cells were not authenticated by us, but retrieved from trusted sources as listed in the Key Resources Table.

#### Mice

Mouse brain tissue used for MATR3 iCLIP was from surplus female C57BL/6 pups sacrificed after birth (P0) and supplied deep-frozen by the animal research facility of the Laboratory for Molecular Biology, Cambridge.

### Method Details

#### siRNA transfection

To deliver siRNAs, Lipofectamin RNAiMax (Life Technologies) was used according to manufacturer’s recommendations. siRNAs are listed in the Key Resources Table.

#### Generation of iCLIP data

iCLIP data for MATR3 and PTBP1 was derived from HEK293T cells, incubated for 8 h with 100 μM 4SU and crosslinked with 2x 400mJ/cm^2^ 365nm UV light. Protein A Dynabeads were used for immunoprecipitations (IP). 80 μl of beads were washed in iCLIP lysis buffer (50 mM Tris-HCl pH 7.4, 100 mM NaCl, 1% NP-40, 0.1% SDS, 0.5% sodium deoxycholate). For the preparation of the cell lysate, 2 million cells were lysed in 1 mL of iCLIP lysis buffer (50 mM Tris-HCl pH 7.4, 100 mM NaCl, 1% NP-40, 0.1% SDS, 0.5% sodium deoxycholate), and the remaining cell pellet was dissolved in 50 μL MSB lysis buffer (50mM Tris-HCl pH 7.4, 100mM NaH2PO4, 7M UREA, 1mM DTT). The mixture was diluted with CLIP lysis buffer to 1000 μl and an additional centrifugation was performed. We found by Western Blotting that up to 50% of MATR3 protein is insoluble by detergent without urea. Lysates were pooled (2ml total volume) and incubated with 4 U/ml of RNase I and 2 μl antiRNase (1/1000, AM2690, Thermo Fisher) at 37°C for 3 min, and centrifuged. We took care to prepare the initial dilution of RNase in water, since we found that RNase I gradually loses its activity when diluted in the lysis buffer. 1.5 mL of the supernatant was then added to the beads and incubated at 4°C for 4 h. The rest of the iCLIP protocol was identical to the published protocol (Huppertz et al., 2014). MATR3 and PTBP1 iCLIP libraries were sequenced on Illumina HiSeq2 machines in a single-end manner with a read length of 50nt.

#### Mapping of iCLIP and eCLIP data

Before mapping the reads, we removed adaptor sequences using the FASTX toolkit version 0.7 and we discarded reads shorter than 24 nucleotides. Reads were then mapped with the iCount suite to UCSC hg19/GRCh37 or mm9/NCBI37 genome assembly using Bowtie v2.0.5 allowing up to two mismatches and up to 20 multiple hits. Unique and multiple mappers were separately analyzed, and to quantify binding to individual loci, only uniquely mapping reads were used. Table S1 lists the source and details including accession numbers of all published iCLIP and HITS-CLIP data used within this study.

The eCLIP libraries were downloaded from ENCODE (Van Nostrand et al., 2017, Sloan et al., 2016). Before mapping the reads, adaptor sequences were removed using Cutadapt v1.9.dev1 and reads shorter than 18 nucleotides were dropped from the analysis. Reads were mapped with STAR v2.4.0i (Dobin et al., 2013) to UCSC hg19/GRCh37 genome assembly. To quantify binding to individual loci, only uniquely mapping reads were used.

To map iCLIP and eCLIP data to the consensus LINE family sequences, adaptor sequences were first removed using custom scripts (for iCLIP) and Cutadapt v1.16 using parameters from the ENCODE eCLIP standard operating procedure (for eCLIP). Reads were then aligned to a custom index generated from L1PA2, L1MA2 and L1ME consensus sequences using Bowtie v1.1.2 with end-to-end mapping, allowing 2 mismatches and unique alignments only. PCR duplicates were collapsed using custom scripts (for iCLIP) and a script from ENCODE (for eCLIP). Alignments in the antisense direction were identified from the SAM flags.

#### TEtranscript estimates of LINE family enrichments

To consider both uniquely mapping and multimapping reads in estimating binding to repeat (sub)families, we used the approach described in TEtranscripts (Jin et al., 2015). In short, for eCLIP FASTQ files, adapters were removed according to the ENCODE eCLIP standard operating procedure. For iCLIP FASTQ files, barcodes were removed using the FASTX-Toolkit (v 0.0.14). For all files, reads aligning to rRNA or tRNA were removed by aligning to custom rRNA and tRNA indices (human or mouse as appropriate) using Bowtie2 (v. 2.2.9, Langmead and Salzberg, 2012). The remaining reads were aligned to the appropriate genome (GRCh38 for human, and GRCm38 for mouse) using STARv2.5.2) with the addition of the parameters “–winAnchorMultimapNmax 100–outFilterMultimapNmax 100” as recommended by TEtranscripts. For each CLIP dataset, TEtranscripts was run using both stranded options (–stranded reverse and–stranded yes) to obtain results for sense and antisense LINE binding.

RNaseq data from ENCODE was used as control, for eCLIP RNaseq of K562 and HEPG2 cells lines (ENCSR885DVH and ENCSR181ZG). For iCLIP samples from mouse brain, we used P2 mouse brain from ENCODE. The iCLIP data in mouse brain was produced from total mouse brain, so we pooled the RNaseq of forebrain, midbrain and hindbrain, accession numbers ENCSR723SZV, ENCSR255SDF and ENCSR749BAG (Sloan et al., 2016).

#### Analysis of PTBP1 binding peaks

PTBP1 iCLIP libraries were pooled, and binding peaks were identified with the iCount suite using randomization based FDR estimates at peak sizes of 3, 15 and 75 nt. cDNA counts in each cluster were normalized and transformed to moderated log2 fold changes with DESeq2, comparing the cDNA count in MATR3-depleted against control samples. We excluded peaks with less than 8 cDNA counts based on inspection of the variability in log2 fold changes of such binding peaks (see Figure 2B).

#### Nucleo-cytoplasmic fractionation for RNA isolation

Cytoplasmic lysis was done as described (Attig et al., 2016) using NP40E-CSK composed of 50 mM Tris-HCl (pH 6.5), 100 mM NaCl, 300 mM sucrose, 3mM MgCl2, 0.15% NP40 and 40 mM EDTA. Cell lysis was allowed to proceed for 5 min on ice, and cytoplasmic supernatant and pelleted nuclei were separated at 4°C, 5000 x g for 3 min. The cytoplasmic supernatant was cleared with two spins (4°C, 5000 x g for 3 min and 4°C, 10000 x g for 10 min). Nuclei were washed with 400μl NP40E-CSK and incubated for 5 min under rotation to ensure complete cell lysis. After repeat of the centrifugation step, nuclei were lysed in 300μl CLIP lysis buffer and sonicated at 5x 30 s pulses in a BioRuptor waterbath device. RNA was isolated using Trizol LS (Invitrogen) and Zymo RNA isolation columns (Zymogen) according to manufacturer’s recommendations. For preparation of RNA for RNaseq, an additional wash step with 180μl NP40E-CSK was done before nuclei rupture.

#### Generation of RNaseq libraries

Before library preparation, purified RNA was DNase I treated for a second time and purified with the DNA-free kit (Ambion). To generate stranded RNaseq libraries, we used the TruSeq stranded RNaseq library kit (Illumina) according to manufacturer’s recommendations; RNA was depleted of rRNA using the RiboZero kit (Epicenter). All libraries were sequenced on Illumina HiSeq2 machines in a single-end manner with a read length of 100 nt.

#### Mapping of RNaseq with TopHat2

Before mapping the reads, adaptor sequences were removed using the FASTX toolkit version 0.7 and we discarded reads shorter than 24 nucleotides. Reads were then mapped with TopHat v2.0.5 (Kim et al., 2013) to UCSC hg19/GRCh37 genome assembly using ENSEMBL version 72 gene annotation as reference, allowing up to two mismatches and only using uniquely mapping hits. RNaseq data files of rRNA depleted cytoplasmic and nuclear RNA from cells depleted of MATR3 and PTBP1 are deposited on EBI ArrayExpress under the accession number E-MTAB-6204.

#### Generation of pAseq libraries and mapping

To quantify poly(A) site usage, we used the QuantSeq mRNA 3′ end sequencing kit (Lexogen) according to manufacturer’s recommendations. We used both the forward and reverse library kit on two independent biological replicates each (four replicates in total). Libraries were prepared from nuclear RNA after individual or combined siRNA depletion of MATR3 and PTBP1/2. All libraries were sequenced on Illumina HiSeq2 machines in a single-end manner with a read length of 100 nt. Poly(A) site usage was analyzed with the expressRNA platform. Reads were trimmed either for adaptor (forward sequencing) or for polyA tails (reverse sequencing strategy) and mapped with STAR v2.4 to UCSC hg19/GRCh37 genome assembly (Dobin et al., 2013), allowing up to 10 mismatches and only using uniquely mapping hits. Since internal priming (i.e., annealing of the oligo-dT primer to a genomic A-rich sequence) is a major problem in 3′ end sequencing protocols, expressRNA removes alignments for which the genomic sequence in the 10 nucleotides upstream and downstream of a polyadenylation event contains stretches of six consecutive A nucleotides or with more than 70% A coverage in any 10-nt window. pAseq raw data is deposited on ArrayExpress at E-MTAB-6287.

#### Semiquantitative RT-PCRs

Reverse transcription was done with 500ng of RNA using RevertAid enzyme (Fermentas) according to manufacturer’s recommendations. The reverse transcription was primed with equal parts of random N6 and N15 oligonucleotides (Sigma) at 100μM concentration. For semiquantitative PCR, we run 35 cycles of amplification with the primer combinations as indicated in each figure (primers are listed in Table S1), and quantified the abundance of each product using Qiaxcel™ (QIAGEN) gel electrophoresis.

#### UV crosslinking assay on recombinant proteins

The RNA probes were made by cloning DNA oligomers into pGEM4Z (Promega) and *in vitro* transcribed and labeled with ^32^P-UTP using SP6 RNA polymerase. We purified full-length N-terminal His-tagged recombinant PTBP1 (rPTBP1) and three MATR3 fragments (rMATR3, amino acids 362-592 or ‘RRMs’, and amino acids 341-592 or ‘RRM-PRI’ with or without mutations in the PRI motif), using Blue Sepharose 6 and HisTrap HP columns. In UV crosslinking assays with recombinant proteins, we used 10fmol of RNA, 0.5μM rPTBP and titrated increasing amounts of rMATR3 fragments against it (0 to 2 μM). After incubation at 30°C for 20 min, the sample was UV cross-linked on ice in a Stratalinker with 1920 milliJoule. The binding reaction was then incubated for 10 min at 37°C together with 0.28 mg/ml RNase A1 and 0.8 U/ml RNase T1. SDS loading buffer was added and the samples heated to 90°C for 5 min before loading on 15% denaturing polyacrylamide gel. To assay binding in HeLa nuclear extract, we prepared standard nuclear extract (Dignam et al., 1983), and combined 10fmol of RNA probe with 0.5 μM rMATR3 and 20% extract.

The ATCTT probe sequence with two embedded AUCUU motifs (shown in bold) and CT-rich stretches in their vicinity (underlined):

GAATACGAATTCCATATATGATCGATAAATATATGGTACCTTGCT**ATCTT**AC**ATCTT**TTTACGGATCCCATATATGATCGATATATATAAGCT.

The CTCTT probe contained six CTCTT motifs (shown in bold):

GAATACGAATTCC**CTCTT**TGAATCGATAA**CTCTT**TGGTACCC**CTCTT**TGATCGATAA**CTCTT**TGGATCCC**CTCTT**TGATCGAT**CTCTT**TAAGCTT

#### Sequence motif analysis

For PTBP1 motifs around iCLIP peaks, we used the strong binding motifs as defined previously (15 pentamers, Haberman et al., 2017), and counted their occurrence around peak centers. To define enrichment, we divided the occurrence at MATR3-dependent and independent peaks by the distribution across all other PTBP1 peaks.

To estimate elements containing putative splice site sequences, we searched for elements with a GGTRAG 5′ SS and a Y_8_NNAGR 3′ SS consensus sequence.

To test for changes in sequence frequency between phylogenetic groups of antisense L1 elements, we calculated the coverage of all 1024 pentamer and 4096 hexamer nucleotide sequences in all L1 elements, normalized by L1 element length, using *maskMotif* (R *Biostrings* package). To interpret these sequence statistics, we matched pentamers with RBP binding motifs, and hexamers with the ESEs and ISSs identified by Fairbrother et al. (2002) and Wang et al. (2013). To identify RBP bindings motifs, we used motifs described in the literature; for PTBP1, TARDBP and HNRNPM, we used the binding motifs that have been validated through functional studies (Gooding et al., 1998, Rot et al., 2017, Xu et al., 2014). For all other proteins, we used RNAcompete motifs (Ray et al., 2013). The number of pentamer motifs per 100 nucleotide gave a distribution for each motif (see Table S7), and we ranked motifs by the difference in the median motif coverage per 100 nt in primate-specific and evolutionarily old L1 elements in which a motif was found. This metric was approximately normal distributed, and we used the 2.5% extremes to obtain an empirical false discovery estimate for motif gain or loss (FDR < 0.05).

To analyze the features of L1 elements with the highest number of binding motifs, we selected the 10% of L1 elements with highest coverage for each of the 13 RBPs. We then compared these with the random expectation based on the total number of each group among all L1 elements. The probability for finding one binding site within 100 nucleotides of random sequence is given in Table S1. Since random expectation for finding a binding site among 100 nucleotides was below 0.25 for all of the RBPs, we considered a 100 nucleotide window as multivalent if motif coverage was more than 10%, corresponding to two pentamer/hexamer motifs. RBPs with such multivalent binding sites within antisense L1 elements are marked in Figure 6C.

#### RNA maps

All metaprofiles of iCLIP data and LINE sequence content around loci of interest (also called RNAmaps) were drawn in R. Metaprofiles are normalized to the number of input loci of each track, and data was smoothed using binning as indicated in figure legends, using the *zoo* package. A generalized script for generation of a metaprofile can be found at https://github.com/JAttig/generalised-Rscripts.

To test for the amount of antisense L1 sequence around MATR3 / PTBP1/2 repressed events in Figure 3A, events significantly increased in absence of either proteins were selected. Misregulated exons are alternative exons selected from splice-array experiments (Coelho et al., 2015), poly(A) site pairs are from mRNA 3′end sequencing experiments. Controls are non-significant events site with no appreciable change (below 10%) and reflect the expected genomic frequency of L1 antisense sequence (shown in gray). Since MATR3 represses exons with significantly larger flanking introns than expected by chance (Coelho et al., 2015), control exons were selected for an identical distribution of intron length.

#### De novo identification of cryptic exons and analysis of differential exon inclusion

In order to predict exons from our RNaseq data, we ran Cufflinks (version 0.9.3, -min-isoform-fraction 0, Trapnell et al., 2012) on the collapsed reads from all cytoplasmic samples of our stranded RNaseq data and then extracted the exons of all predicted transcripts. After flattening the Cufflinks output to non-overlapping exonic bins, our Cufflinks prediction contained 671,956 exonic bins. Next, we estimated exon inclusion using Whippet (Sterne-Weiler et al., 2017). Neighboring exonic bins with equal inclusion levels were merged. We only considered exonic bins of at least 5 nucleotides in size, supported by at least six reads, and estimated inclusion level above 15% in control or test condition for analysis (165,138 exonic bins).

All exons that were not identical with exons annotated in UCSC gene annotation (hg19) were referred to as ‘cryptic’. Table S3 shows a complete breakdown of the annotation of exonic bins. For readability, we refer to ‘exonic bins’ as ‘exons’ throughout the text. To annotate LINE-derived exons previously known to be alternatively spliced, we used the ‘knownAlt Events’ and ‘knownGene’ from UCSC TableBrowser for hg19, downloaded on 28^th^ March 2014. In addition, we downloaded the ‘refGene’ table on 23^rd^ March 2017. All exons annotated by UCSC were collapsed within a gene to unique exonic ranges, and classified as alternative or constitutive exon as follows. All exons not annotated as alternative by UCSC and present in the RefSeq exon annotation with identical genomic coordinates were classified as constitutive, all other exons were considered alternative exons.

Differential splicing of exons was called using Whippet’s probability estimate with a cut-off of 0.85 in either of the two MATR3/PTBP1 depletion conditions. All exons with one or both splice sites residing within a LINE repeat (as annotated by RepeatMasker, Smit et al., 1996–2004) were assigned as LINE-exons.

#### Analysis of LINE-derived exon inclusion in human tissues

To analyze inclusion of exons across human tissues, we used data on mapped junctions from the V6p release of the GTEx Consortium (2015) (https://www.gtexportal.org/home/, dbGap accession phs000424.v6.p1). We used UCSC/RefSeq annotation (see above) and isolated all LINE-derived exons as well as Alu-exons. Then, we selected all exons from genes with at least one Alu- or LINE-derived exon. We identified junction-spanning reads to each of these exons in a 2 nt grace window around the splice site and used those to identify the 5′ and 3′ splice site of the upstream and downstream exon. We identified internal exons by restricting the data to exons with upstream and downstream junctions. We only allowed a single exon inclusion isoform across tissues (i.e., identical flanking exons) and chose the isoform with more junction reads. To ensure sequencing depth and gene expression were sufficient to calculate exon inclusion, we only used exons with at least 200 reads across the 8,555 samples (average of up+downstream junctions or skipping junctions). We calculated the Percent-spliced-in as

PSI=50∗(upstream+downstreamjunctions)/(skippingjunction+0.5∗(upstream+downstreamjunctions)),

and inclusion within each tissue as average of all samples. If an exon was absent in any tissue, as judged by absence of any junction spanning read and any read for the skipping junction, it was treated as ‘data not available’ for this particular tissue. In total, we covered 43583 exons across 52 tissues and sub-tissues, which were adipose tissue (sub-cutanoues and visceral omentum), adrenal glands, artery (aorta, tibial and coronary artery), bladder, brain, breast, cervix (ecto- and endo-cervix), colon (sigmoid and transverse), esophagus (mucosa, muscularis and gastresophageal junction), fallopian tube, heart (atrial appendage and left ventricle), kidney (cortex), liver, lung, skeletal muscle, nerve tissue (amygdala, anterior cingulate cortex, caudate basal ganglia, cerebellar hemisphere, cerebellum, cortex, frontal cortex, hippocampus, hypothalamus, nucleus accumbens basal ganglia, putamen basal ganglia, cervical spinal cord, substantia nigra, tibial), ovary, pancreas, pituitary, prostate, minor salivary gland, small intestine terminal ileum), spleen, skin (suprapubic and lower leg), stomach, thyroid, testis, uterus and vagina, as well as EBV transformed lymphocytes and transformed fibroblasts. We did not use data from whole blood, which had poor coverage on most genes. On top of the PSI index for each tissue, we collated the data across tissues and computed the maximum difference in PSI between the tissue(s) with highest inclusion and lowest inclusion of each exon. Because testis is known to be a very promiscuously transcribed tissue and accordingly showed many LINE-derived exons exclusively observed in the testis, we only included exons which showed at least 5% inclusion in any tissue, except testis.

Within the LINE-derived exons, we were surprised to find that L1-derived exons are a rich source of exons in the regions of the genome that encode the highly variable and species-specific immunoglobulin variable chain region (the Ig-region on chromosomes 2, 14, 15, 16 and chr22). The Ig-domain containing proteins are among the most quickly evolving genes within mammals and the sequence of Ig-regions is highly species-specific, including a particular richness in lineage-specific repeats (Hughes, 1997, Sepulveda et al., 2005). We find the human region is densely packed with 1,845 LINEs, 1,152 of which produce exons according to exon annotation by UCSC. The LINE-derived exons in these regions are almost exclusively seeded by primate-specific L1s. However, we consider them as cryptic exons, since we did not detect them by our analysis of the GTEx data and their average splice site score was several orders of magnitude below other LINE-derived or known alternative exons. Hence, we assume many of these exons are mistakenly annotated as exons, as a consequence of the repetitive nature and recombination events at this locus. For this reason, we ignored all LINE-derived exons from the Ig-regions in our analysis. However, the Ig-regions might be an unusual exception, where exonisation of repeat sequences is not under negative selection pressure, due to the need to generate protein-diversity, and because B- and T cell selection ensures only cells with a functional protein survive. Detailed analysis of B and T cell receptor sequences after Ig-locus recombination will be needed to further examine the contribution of these young L1-derived exons to the expression of immunoglobulin genes.

#### Annotation of ‘established’ alternative exons in mouse and human

For annotation of lowly or highly included alternative exons in human, we used the data on mapped junctions from the V6p release of the GTEx Consortium (2015) (https://www.gtexportal.org/home/); as above, with minor differences. We limited the exon set to internal exons with minimum 500 supporting junction reads, and calculated the average PSI value across all tissues excluding testis, vagina and EBV transformed cell lines. We considered as constitutively included exons those with average inclusion above 85%, as alternative those with average inclusion between 15% and 85%, and as lowly included those with an average below 15%.

For annotation of mouse exons, we used the annotation provided by Merkin et al. (2015), which analyzed RNaseq data from three individual mice. We considered as alternative exons those which are alternative in all individuals (i.e., below 97% inclusion in at least one tissue), and as constitutive exons those which are constitutive in all individuals. We discarded exons which are heterogeneous between individuals. The exon set is annotated in Merkin et al. (2015), Table S2.

#### Classification of repeat element age by divergence or phylogenetic tracing

To compare the divergence of LINE insertions from their consensus sequence, we used the nucleotide difference / 1000nt, which is provided for each repeat element by the RepeatMasker table (hg19, Repeat Library 20090604, (Smit et al., 1996–2004)).

For phylogenetic tracing, we tested for presence of orthologs positions with the UCSC Genome Browser LiftOver tool, using the respective all-chain BLASTZ files. Human and mouse LINE repeats from hg19 and mm9 RepeatMasker annotation were first lifted to hg38 and mm10. We then tested for the presence of each LINE repeat in the human and mouse lineage by retrieving ortholog genomic loci for the genomes of rhesus macaque (rheMac8), gorilla (gorGor5), mouse (mm10), rat (rn6), dog (canFam3) and cow (bosTau8). To curate the LiftOver results and safeguard against misannotation by errors in the genome lift, we cross-referenced for all liftover positions if the element overlaps with a LINE annotated by RepeatMasker for the respective genome, and only refer to the element as present in a species if at least 33% of the lifted genomic position are LINE-derived as annotated by RepeatMasker. All other elements are either ‘notLINE’ if they were not identified by RepeatMasker, ‘degenerate’ if LiftOver reported them as ‘partially-deleted’, or ‘absent’ if LiftOver reported them as ‘deleted’. Elements from hg19 that were not ‘present’ in hg38 were discarded entirely. Then we converted the LiftOver annotation to phylogenetic groups after manual inspection of the liftover results in the following manner. We denoted elements as human- and primate-specific, which are ‘absent’ in all other species. We denoted additional elements as primate-specific, if they were either ‘present’, ‘degenerate’ or ‘notLINE’ in at least one of the two primate species, and ‘absent’ or ‘notLINE’ in all of the others. We denoted elements as specific for the euarchontoglires branch, if the element was ‘absent‘ or ‘notLINE’ in the two laurasiatherian species, and ‘present’ or ‘degenerate’ in mouse or rat. The remaining elements were all lifted toward at least one of the two laurasiatherian species, and hence present in the last common ancestor of the species we surveyed. Elements present in one but absent in the other were denoted as found in ‘one distant species’, elements present in both as found in ‘two distant species’. All remaining elements were either reported as degenerate in both species, or the liftover results were ‘unclear’ (for example if the element was lifted to many species but did not overlap with the LINE annotation in any of those). In either case, we ignored the corresponding element for phylogenetic comparisons and all analysis. Group sizes for L1 elements in the hg19 assembly were:

**Table:** 

Primate-specific LINE insertions	459,702
Euarchontoglires-specific insertions	38,642
One-distant species	113,263
Two-distant species	29,476
Sequence degenerated elements	130,949
unclear liftover results	179,832

These are listed in Table S5.

#### Calculation of a normalized binding score for RBPs on LINE fragments

To compare binding preferences of RBPs between different groups of LINEs, we calculated a binding score for each RBP on each LINE as follows. To ensure that we assessed elements that are part of expressed transcripts, we selected the 10% of L1 elements with highest coverage by any of the 121 RBPs. All phylogenetic groups were represented in this selection in expected proportions. Next, we averaged the binding of each RBP against the sum of all RBPs, generating a relative binding metric among all RBPs (ranging from 0 to 1). We then visualized any preferences in binding to a phylogenetic group as enrichment by normalizing to the mean between the groups.

#### Comparison of exon-proximal and deep intronic antisense L1 elements

We tested the distance of antisense L1 elements more than 100 nucleotides in size to the exons for which we calculated inclusion from GTEx. We considered as ‘exon-proximal’ elements within 500 nucleotides of a constitutively used exon, and as ‘deep-intronic’ elements that are more than 2000 nucleotides from any exon.

To align their sequences against L1 consensus, we selected L1ME, L1MA2 and L1PA2 family consensus sequences from repBase. The entry of L1PA2 was missing the L1 5′ UTR, and we chose the L1PA10 sequence to complement it. We aligned all elements against a blast database using blastn (Camacho et al., 2009), and kept for each element the best alignment. Blast settings were ‘-strand minus -soft_masking FALSE -evalue 2 -word_size 13’. In total, we aligned 33,751 L1 elements considered as ‘deep-intronic’ and 10.655 L1 elements considered as ‘exon-proximal’.

### Quantification and Statistical Analysis

#### Sample size and replicates

Whenever referred to in the text, *replicates* stands for biological replicates, defined as samples collected independently of one another in separated experiments. In case of the iCLIP experiments from MATR3 or PTBP1 depleted cells, sequencing files were pooled across 2 biological replicates because coverage varied widely within them, and only the pooled data was used.

#### Software and Statistics

All statistical analyses were performed in the R software environment (version 3.1.3 and 3.3.2, https://www.r-project.org) or in PRISM6 (GraphPad Software). Key software used in analysis of high-throughput sequencing data are listed in the Key Resources Table.

Sample size and statistical tests are provided in the figure and figure legends. We generally made use of nonparametric tests because data distributions failed to conform with the assumption of normality and equal variance (homoscedasticity), assessed visually with qqnorm plots. The only data analyzed by parametric tests are semiquantitative RT-PCR assays (shown in Figure 4), here one-way ANOVA was used coupled with multiple comparison correction for pairwise comparisons according to Tukey’s HSD.

### Data and Software Availability

#### Data

The accession number for the RNA-seq data files of rRNA depleted cytoplasmic and nuclear RNA from cells depleted of MATR3 and PTBP1 reported in this paper is EBI ArrayExpress: E-MTAB-6204. The accession number for the mRNA 3-end sequencing files of cells depleted of MATR3 and PTBP1 reported in this paper is EBI ArrayExpress: E-MTAB-6287. The accession number for the iCLIP sequencing data files of PTBP1 reported in this paper is EBI ArrayExpress: E-MTAB-6286. The accession number for the iCLIP sequencing data files of MATR3 reported in this paper is EBI ArrayExpress: E-MTAB-6267 (human cells) and E-MTAB-6283 (mouse brain). These and published datasets referenced throughout this study are listed for convenience in Table S1, including accession details.

## Supplemental Information

Table S1. Sources and References for CLIP and RNA-Seq Datasets, as Well as RBP Binding Motifs, Related to STAR Methods This file contains 6 spreadsheets (tabs) in total, all dedicated to provide the source of data used to generate the main figures; including accession number of published iCLIP, eCLIP and RNaseq data. Tab 4 contains the motifs we considered high-affinity binding motifs of PTBP1 (Figure 2), and Tab 5 contains references to trace which RBP we presumed as potential binding partner of the pentamer motifs analyzed in Figure 6.

Table S2. Quantification of L1 and L2 Sequences in iCLIP and eCLIP, Related to Figure 1 This table contains the percentage of reads we found to map to L1 and L2 sequences in sense or in antisense in each data set.

Table S3. Summary Statistics of Cryptic Exon Annotation Based on UCSC Annotation and Cufflinks Assembly, Related to Figure 3 This table explains our definition of cryptic exons, and the number of LINE-derived exons at 15% inclusion level.

Table S4. Summary Statistics of mRNA 3-End Sequencing Experiments, Related to Figure 3 This table summarizes the analysis of changes in polyA site usage (pairs of pA1 and pA2), and if any of the two polyA sites originates from inside a LINE repeat.

Table S5. Annotation Derived from Phylogenetic Tracing of L1 Elements in hg19, Related to Figure 5 The table contains the position and result of cross-species comparison for all RepeatMasker annotated L1 elements in hg19. The first eleven columns correspond to the information provided by RepeatMasker. Then 4 columns provide annotation of each L1 as used in this publication. The remaining columns refer to the individual result from liftover of the L1 to the respective genome (hg38, gorGor5, rheMac8, mm10, rn6, bosTau8, canFam3), and consists of one of four categories: absent, degenerate, notLINE, or present. Degenerate means the L1 was lifted to the genome, but was heavily truncated (size less than 33% of human). notLINE means the L1 was lifted but the liftover position is not recognized as a LINE repeat by RepeatMasker in the new genome.

Table S6. Inclusion Levels of 43583 UCSC Annotated Exons in 53 Human Tissue Types, Related to Figure 5 Repeat-derived exons were defined as any exon, for which either the 5′ or the 3′ splice site is derived from an Alu or a LINE repeat. We quantified these and all exons of the same genes in the V6p data of the GTEx consortium. In total, we covered 43583 exons across 53 tissues and sub-tissues. The data is from hg19 and positions are 0-based.

Table S7. Summary Statistics of Pentamer Frequencies in Antisense L1 Sequences, Related to Figure 6 This table provides summary statistics on the frequency of every pentamer within antisense L1 repeats. Column 1 contains the pentamer itself, column 2 the percentage of repeats that have at least one occurrence of the sequence, column 3 the median count in primate-specific elements as percent of 100nt sequence (excluding 0 counts). Column 4 is the difference in the median count of primate-specific versus old L1 insertions, and column 5 the rank based on column 4. Column 6 specifies the possible binding partner of the pentamer, if known.
