# Supplementary material for: Heteromeric RNP Assembly at LINEs Controls Lineage-Specific RNA Processing
Source: Cell. 2018 Aug 23;174(5):1067–1081.e17. doi: 10.1016/j.cell.2018.07.001 (PMC6108849; doi:10.1016/j.cell.2018.07.001)
Supplement: Table S3. Summary Statistics of Cryptic Exon Annotation Based on UCSC Annotation and Cufflinks Assembly, Related to Figure 3 — This table explains our definition of cryptic exons, and the number of LINE-derived exons at 15% inclusion level. [file mmc3.pdf]

Supplementary Table 3. Related to Figure 3.

Cufflinks predicted exonic bins*

*Cufflinks prediction was flatted from gtf to gff for HTseqcounts.py

exonic bins of min 5nt length, min 6 reads coverage

referenced to UCSC

	total	truly novel exons	LINE-derived
exonic bins	183,361	32,737	3,811
# of genes	9,958	7,604	2,241
with min 15% inclusion in control or MATR3/PTBP1 depleted cells (Whippet)			
exonic bins	149,718	26,717	1,702
# of genes	9,957	7,298	1,085

LINE-derived exons<br>total: 1,702			"cryptic exons"	
UCSC annotation:	constitutive	alternative	partial-overlap	truly novel
	211	311	875	305
