# Supplementary material for: Heteromeric RNP Assembly at LINEs Controls Lineage-Specific RNA Processing
Source: Cell. 2018 Aug 23;174(5):1067–1081.e17. doi: 10.1016/j.cell.2018.07.001 (PMC6108849; doi:10.1016/j.cell.2018.07.001)
Supplement: Table S4. Summary Statistics of mRNA 3-End Sequencing Experiments, Related to Figure 3 — This table summarizes the analysis of changes in polyA site usage (pairs of pA1 and pA2), and if any of the two polyA sites originates from inside a LINE repeat. [file mmc4.pdf]

Supplementary Table 4. Related to Figure 3.

changes in pA-site usage

condition	proximal pA site usage	FDR	#	# within LINE repeats proximal	# within LINE repeats distal
Matr3 KD1	up	< 0.05	257	9	6
		>= 0.05	417	24	24
	down	< 0.05	329	26	5
		>= 0.05	428	54	22
	neither	-	2,605	150	63
Matr3 KD2	up	< 0.05	214	11	7
		>= 0.05	416	41	27
	down	< 0.05	368	14	5
		>= 0.05	567	46	26
	neither	-	2,256	131	56
PTB KD	up	< 0.05	219	5	4
		>= 0.05	420	28	21
	down	< 0.05	401	18	4
		>= 0.05	516	52	28
	neither	-	2,332	139	65
Matr3 KD1 + PTB KD	up	< 0.05	370	17	11
		>= 0.05	424	34	21
	down	< 0.05	399	24	5
		>= 0.05	492	44	28
	neither	-	2,373	129	59
